# Glucosinolate Derivatives: Emerging Anti-Inflammatory Agents

**DOI:** 10.3390/ph19050658

**Published:** 2026-04-22

**Authors:** Sandrine Ressurreição, Sónia A. Pinho, Maria Teresa Cruz, Lígia Salgueiro, Artur Figueirinha

**Affiliations:** 1 University of Coimbra, Faculty of Pharmacy, 3000-548 Coimbra, Portugal; sandrine@esac.pt (S.R.); spinho@cnc.uc.pt (S.A.P.); trosete@ff.uc.pt (M.T.C.); amfigueirinha@ff.uc.pt (A.F.); 2 Polytechnic University of Coimbra, Coimbra Agriculture School, 3045-601 Coimbra, Portugal; 3 Chemical Engineering and Renewable Resources for Sustainability (CERES), University of Coimbra, 3030-790 Coimbra, Portugal; 4 Center for Innovative Biomedicine and Biotechnology (CIBB)/Center for Neuroscience and Cell Biology (CNC), University of Coimbra, 3004-504 Coimbra, Portugal; 5 Associated Laboratory for Green Chemistry (LAQV) of the Network of Chemistry and Technology (REQUIMTE), University of Coimbra, 3000-548 Coimbra, Portugal

**Keywords:** glucosinolate derivates, anti-inflammatory activity, NF-κB, NRF2, MMPs

## Abstract

Glucosinolates are sulfur-containing secondary metabolites predominantly found in Brassicaceae plants, which, upon enzymatic hydrolysis, generate bioactive compounds with potent anti-inflammatory properties. These derivatives modulate key inflammatory pathways by inhibiting NF-κB nuclear translocation, reducing pro-inflammatory cytokine production, including TNF-α, IL-6, and IL-1β, and suppressing iNOS and COX-2 expressions. They also activate NRF2-dependent antioxidant defenses, upregulating enzymes such as HO-1 and NQO1, and regulate MMPs, contributing to tissue protection during chronic inflammation. Evidence from in vitro and in vivo studies consistently demonstrates their ability to attenuate inflammation and oxidative stress. Although approximately 137 glucosinolates have been identified, only about twelve have been investigated in detail regarding the anti-inflammatory activity of their derivatives, highlighting a significant gap in current knowledge and considerable potential for the discovery of new therapeutic compounds. In this context, a systematic survey was conducted of plant species reported in scientific literature as sources of glucosinolates, with particular emphasis on studies evaluating their extracts and fractions for anti-inflammatory potential in in vitro and in vivo experimental models. Additionally, this review also aims to highlight the anti-inflammatory and antioxidant potential of glucosinolate-derived compounds, focusing on their modulation of the NF-κB and NRF2 signaling pathways and their ability to regulate matrix metalloproteinases. It also emphasizes that, despite the broad diversity of glucosinolates identified to date, only a limited number have been functionally investigated. By addressing this gap, and based on the systematic survey performed, this review underscores the need for further research to fully explore their therapeutic potential.

## 1. Introduction

Glucosinolates are sulfur-containing secondary metabolites that play a key role in plant chemical defense against herbivores and pathogens [1,2]. They are particularly abundant and structurally diverse in the Brassicaceae family, although they also occur in other families within the order Brassicales, typically at lower concentrations and with distinct chemical profiles. Among these families, Capparaceae (e.g., *Capparis spinosa*), Cleomaceae (e.g., *Cleome spp.*), Caricaceae (e.g., *Carica papaya*), Tropaeolaceae (e.g., *Tropaeolum majus*), Resedaceae (e.g., *Reseda lutea*), and Moringaceae (e.g., *Moringa oleifera*) contain glucosinolates or related sulfur-containing derivatives [2,3,4,5,6,7,8]. These compounds confer unique chemical, nutritional, and functional properties to species within these families and are a major focus of scientific research.

Glucosinolates exhibit relatively limited biological activity in their intact form, but when plant tissue is damaged, they undergo enzymatic hydrolysis by myrosinase, producing an unstable aglycone. This intermediate can then rearrange into a variety of biologically active compounds, such as isothiocyanates, nitriles, thiocyanates, oxazolidine-2-thiones, epithionitriles, indole-3-carbinol (I3C) and 3,3′-diindolylmethane (DIM). The specific profile of these hydrolysis products is strongly influenced by the chemical structure of the glucosinolate side chain, as well as by environmental factors like pH and the presence of specific proteins [9,10,11,12,13,14]. These hydrolysis products are of particular interest due to their bioactivity, including anti-inflammatory effects mediated by modulation of pro-inflammatory signaling pathways and reduction in cytokine production [15,16,17,18].

This review focuses on the anti-inflammatory properties of glucosinolates and their derivatives, while also providing a comprehensive and up-to-date overview of their biosynthetic pathways, classification, hydrolysis products, extraction, and analytical methods. It synthesizes current evidence on their mechanisms of action and underscores their relevance as natural modulators of inflammatory processes. Additionally, it highlights existing knowledge gaps, emphasizing the need for further studies to fully elucidate their therapeutic potential. What distinguishes this article from previous reviews is its specific focus on the anti-inflammatory potential of glucosinolate derivatives, summarizing current evidence on their mechanisms of action and highlighting their relevance as natural modulators of inflammation, while also emphasizing that many aspects remain underexplored, indicating the need for further research to fully understand their therapeutic potential.

## 2. Methodology

For the present review, we collected papers published from 1 July 1982 to 28 February 2026 and available in scientific electronic databases, including Web of Science, Science Direct, PubChem, PubMed, Google Scholar.

## 3. Structure, Classification and Biosynthesis of Glucosinolates

**Figure 1:**
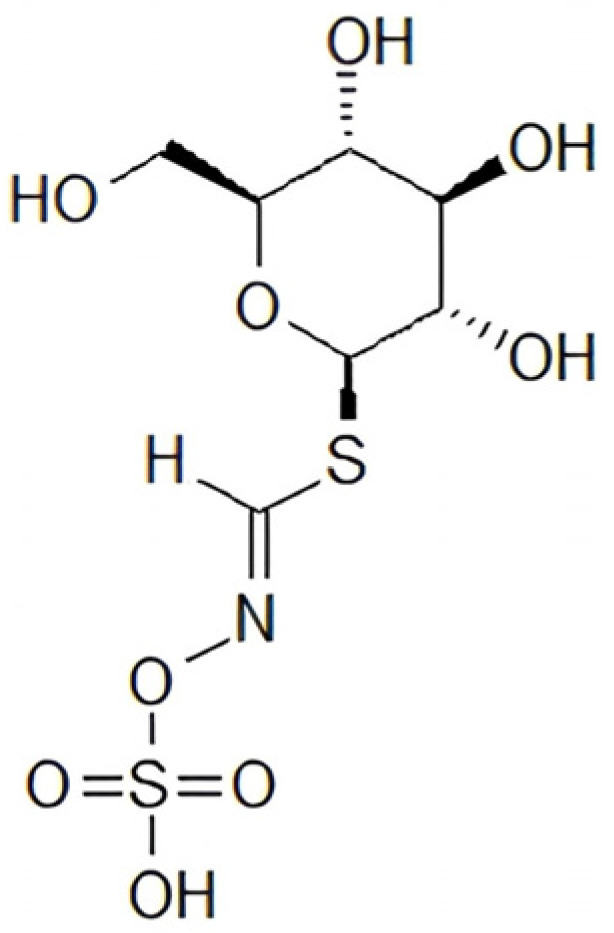
General structure of glucosinolates.

Glucosinolates, previously referred to as thioglucosides, are sulfur-containing compounds composed of a *β*-D-thioglucose moiety linked to a sulfonated oxime group and a variable side chain (R) derived from an amino acid (Figure 1) [19,20,21].

**Figure 2:**
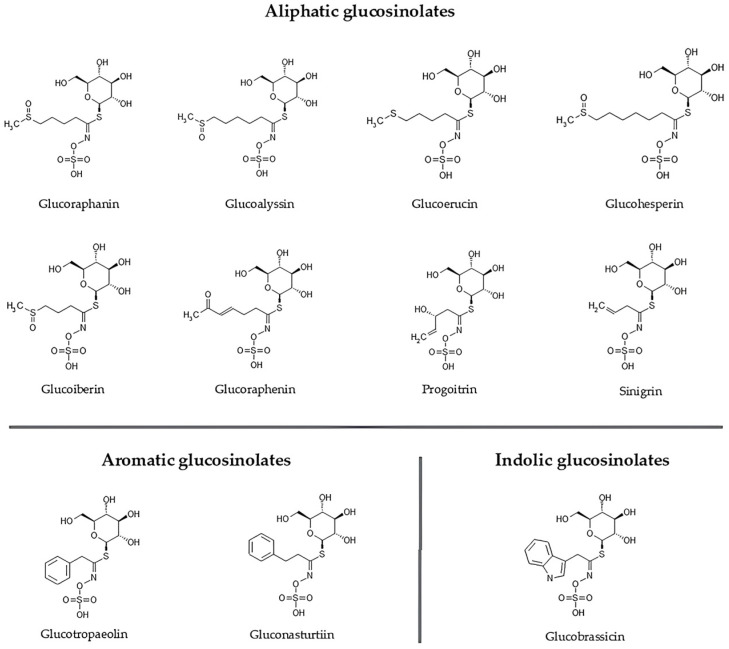
Classification of glucosinolates based on their amino acid precursors, with examples for each class: aliphatic, aromatic, and indole glucosinolate.

Glucosinolates are biosynthesized from amino acids, so plants containing these compounds inherently possess the corresponding amino acids as key metabolic precursors [22,23]. Their structural diversity and biological activity are determined by the amino acid-derived side chains, which classify glucosinolates into three main groups: aliphatic, indole, and aromatic (Figure 2). Aliphatic glucosinolates, primarily derived from methionine and, to a lesser extent, from valine, leucine, and isoleucine, are the most abundant and play a central role in plant defense against herbivores. Indolic glucosinolates, synthesized from tryptophan, contribute to plant growth regulation, stress responses, and pathogen defense. Aromatic glucosinolates, originating from phenylalanine or tyrosine, are less common but enhance chemical diversity and support a broad spectrum of defensive strategies [19,24].

**Figure 3:**
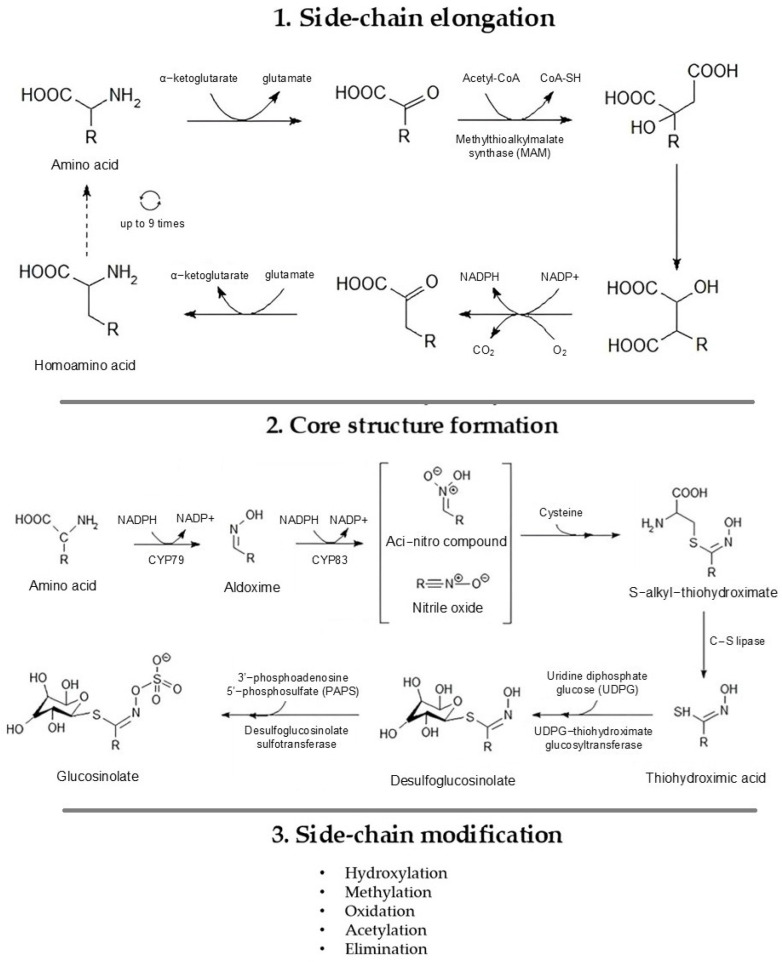
Biosynthesis of glucosinolates.

The biosynthesis of glucosinolates occurs through three main stages: side-chain elongation, formation of the core glucosinolate scaffold, and side-chain modification (Figure 3). The first stage, side-chain elongation, is particularly important because the chemical nature of the side chain largely determines the structure of the hydrolysis products. This process begins with the transamination of the precursor amino acid, followed by condensation reactions, isomerization, and oxidative decarboxylation, ultimately producing the elongated amino acid derivative. In the second stage, the core glucosinolate structure is assembled through a series of five enzymatic and non-enzymatic transformations. Initially, the elongated amino acid is converted into an aldoxime by cytochrome P450 enzymes of the CYP79 family. This aldoxime is then oxidized by CYP83 enzymes to form an unstable nitrile oxide intermediate. These intermediates are further converted non-enzymatically into alkylthiohydroximic acids, which, in the presence of C–S lyase, yield thiohydroximic acids. Subsequent S-glycosylation by UDP-glucose-dependent thiohydroximic glucosyltransferase produces desulfoglucosinolates, and the final sulfation by desulfoglucosinolate sulfotransferase generates the fully formed glucosinolate core structure. The final step involves side-chain modifications of glucosinolates, including oxidation, hydration, and acetylation reactions, catalyzed by specific enzymes such as CYP79 and CYP83 (oxidation), sulfotransferases (SOTs) and glucosyltransferases (for conjugation and stabilization), and acetyltransferases (for acetylation), leading to the formation of the final functional glucosinolate [25,26].

The biosynthesis of glucosinolates is influenced by the type of precursor amino acid. While all pathways involve core structure formation and subsequent side-chain modifications, differences in chain elongation and the chemical nature of the precursor amino acid are primary factors driving the structural diversity of glucosinolates. Aliphatic glucosinolates, derived from amino acids such as methionine, leucine, isoleucine, valine, alanine, and glutamate, often begin with chain elongation, where the carbon chain of the amino acid is extended. This is followed by the formation of the core glucosinolate structure, which consists of a sugar-linked sulfate group and a cyanate moiety. The final step involves side-chain modifications, including reactions such as oxidation, hydration, or elimination, which create chemical diversity among aliphatic glucosinolates. Benzenic glucosinolates, originating from aromatic amino acids like phenylalanine and tyrosine, undergo chain elongation only in the case of phenylalanine; tyrosine does not experience this step. Afterward, the core structure is formed, and the side chains are modified, through processes like acetylation or hydration, generating a variety of aromatic glucosinolates. Indolic glucosinolates, derived from tryptophan, do not undergo chain elongation; their biosynthesis involves only the formation of the core structure and side-chain modifications, producing compounds important for defense and signaling. Overall, while all glucosinolate pathways share core formation and side-chain modification, differences in chain elongation and the type of precursor amino acid determine the structural diversity of the glucosinolates [7,26].

Although intact glucosinolates exhibit relatively limited biological activity, their hydrolysis products have attracted considerable interest due to their potent pharmacological properties [7,27]. These plant secondary metabolites are precursors of bioactive molecules that are fundamental for plant protection against both biotic and abiotic stresses. Against biotic stress, caused by herbivores or pathogens, they can act directly to deter attackers or indirectly by activating defense responses. In the case of abiotic stress, such as drought, salinity, extreme temperatures, or radiation, they contribute to plant survival by enhancing antioxidant activity, regulating stress-responsive genes, and maintaining cellular balance. Through these functions, they support plant growth, development, and resilience under adverse conditions [28,29].

**Figure 4:**
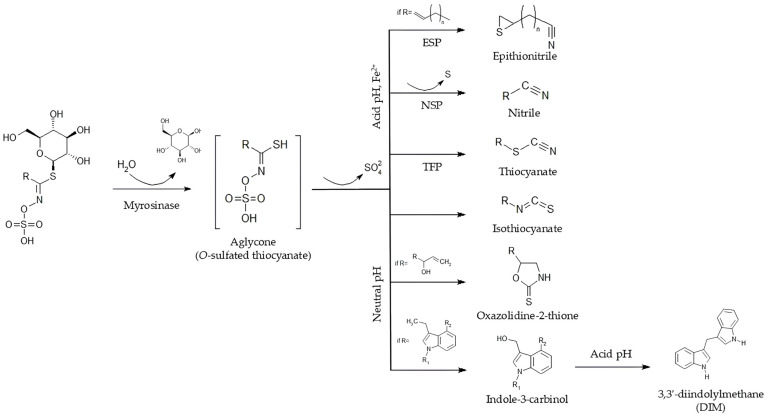
Glucosinolates and their hydrolysis products.

Glucosinolate degradation occurs primarily through the action of the enzyme myrosinase, which is typically compartmentalized in vacuoles or enzymatic granules separate from the glucosinolates, which are themselves stored in other vacuoles or in the cytoplasm depending on the species and type of glucosinolate [27,30]. This segregation prevents premature hydrolysis, avoiding the formation of toxic compounds while the cell remains intact, a phenomenon widely referred to as the “mustard oil bomb” defense system [27,30,31,32]. When plant tissues are damaged by herbivores, pathogens, or mechanical injury, myrosinase interacts with glucosinolates, promoting the formation of reactive compounds such as isothiocyanates, thiocyanates, nitriles, epithionitriles, oxazolidine-thiones, I3C and DIM [27,30,33] (Figure 4). These products act as toxic or repellent agents, inhibiting herbivore feeding and growth, restricting pathogen development, and suppressing the growth of competing plants [30,33]. Furthermore, they function as chemical signals that activate systemic defensive responses in unaffected tissues, preparing healthy cells to withstand future attacks [28,29,34].

The products of glucosinolate hydrolysis are diverse. The hydrolysis of a glucosinolate initially forms an unstable aglycone (*O*-sulfated thiocyanate), which can rearrange to form isothiocyanates, thiocyanates, nitriles, epithionitriles, oxazolidine-thiones, indole-3-carbinol (I3C) and 3,3′-diindolylmethane (DIM), as well as inorganic sulfates and glucose [35,36]. Isothiocyanates, which predominate under neutral pH conditions and in the absence of specific cofactors, are recognized as the main bioactive products of glucosinolates due to their potent anticancer properties, as well as their anti-inflammatory, antioxidant, and antimicrobial activities [36,37,38]. Isothiocyanates, such as sulforaphane and allyl isothiocyanate, have been highlighted for their strong biological effects [37,39].

Another relevant bioactive class arises from indolic glucosinolates, formed only when the R group is indole, as in glucobrassicin. During digestion, these compounds generate I3C, which can subsequently be converted into DIM, as the indole side chain disfavors the typical rearrangement of the unstable aglycone into isothiocyanates, thereby directing the hydrolysis pathway toward the formation of indole-derived compounds [38,40]. I3C and DIM exhibit multiple bioactivities, including modulation of estrogen metabolism, support of detoxification, reduction in inflammation, antioxidant activity, regulation of apoptosis, and influence on immune function, highlighting their potential role in disease prevention and overall health [41]. The anti-inflammatory activity of these compounds will be discussed in detail in Section 6 of this article.

Nitriles, thiocyanates and epithionitrile form more readily at acidic pH in the presence of ferrous ions or in the presence of specific proteins, such nitrile-specifier proteins (NSP), thiocyanate-forming protein (TFP) and epithiospecifier protein (ESP), and although they are less potent, some exhibit antimicrobial activity [35,39]. Another group includes oxazolidine-2-thiones, such as goitrin, primarily formed from glucosinolates with hydroxyalkenyl side chains, which interfere with thyroid hormone synthesis and have been reported to exert a mild inhibitory effect on NF-κB signaling, although this activity is considerably weaker than that of isothiocyanates [35,39].

In addition to plant myrosinase, glucosinolate hydrolysis can also be mediated by microorganisms in the gastrointestinal tract [42,43,44]. The intestinal microbiota contributes to the conversion of glucosinolates into bioactive compounds through specific gut microorganisms, such as *Bacteroides thetaiotaomicron*, species of *Lactobacillus* and *Bifidobacterium*, as well as other bacteria that possess myrosinase, the enzyme responsible for hydrolyzing glucosinolates into isothiocyanates and other beneficial bioactive derivatives. [20,45]. This microbial conversion becomes especially important when cooking inactivates the plant’s own myrosinase, allowing intact glucosinolates to reach the colon where microbial myrosinase can act [20,45]. The efficiency of this process depends on the individual’s gut microbiome composition, enzyme activity, and interactions with other dietary components, all of which influence the bioavailability and health-promoting effects of the resulting compounds in vivo [46]. Hydrolysis products are rapidly absorbed in the small intestine, primarily through passive diffusion. After absorption, isothiocyanates enter cells and exert their main biological activity in their free form [21,46]. Concurrently, they are swiftly metabolized through the mercapturic acid pathway. In this process, the isothiocyanate initially reacts with glutathione in a reaction catalyzed by glutathione S-transferase enzymes, forming an isothiocyanate–glutathione conjugate. This conjugate subsequently undergoes sequential enzymatic transformations, generating cysteinylglycine, cysteine, and ultimately *N*-acetylcysteine conjugates [21,46]. During these metabolic steps, partial reversibility may occur, allowing the release of small amounts of free isothiocyanate, with the conjugates acting as transient intracellular reservoirs. However, the final metabolites exhibit limited or no biological activity [47]. The end product, the *N*-acetylcysteine mercapturic acid derivative, which lacks significant biological activity, is transported to the kidneys, where it is predominantly excreted in the urine, representing the principal route of elimination of these compounds from the body [21,46,47,48,49].

The structure–activity relationships (SAR) of glucosinolates are strongly influenced by their side-chain structure and their enzymatic conversion into bioactive degradation products, particularly isothiocyanates [6]. Aliphatic-, aromatic-, and indolic-derivate isothiocyanates all share the electrophilic –N=C=S group, which reacts with protein thiols such as cysteine residues, modifying regulatory proteins including Keap1 and transcription factors like NF-κB. This covalent interaction activates Nrf2-mediated antioxidant and phase II detoxifying enzymes, modulates MAPK and apoptosis pathways, and suppresses pro-inflammatory cytokines, underpinning their chemopreventive, anti-inflammatory, and cytoprotective effects. Structural variations determine specific activity: aliphatic-derivate isothiocyanates, with longer or more hydrophobic side chains, enhance membrane permeability and cellular uptake; aromatic-derivate isothiocyanates are more chemically stable and potent in inducing apoptosis, cell cycle arrest, and immune modulation; and indolic-derivate isothiocyanates particularly influence hormone-related and epigenetic pathways, effectively reducing inflammatory signaling in hormone-sensitive tissues. Collectively, these SAR insights illustrate how isothiocyanates achieve their diverse chemopreventive, anti-inflammatory, and cytoprotective functions [18,47,50,51].

## 4. Extraction and Analysis of Glucosinolates and Their Hydrolysis Products

The identification of glucosinolates in plant matrices is analytically challenging due to their similar structure and polarity. These characteristics complicate chromatographic separation, accurate identification, and reliable quantification, particularly in complex plant extracts [52,53,54]. To address these limitations, a variety of analytical methodologies have been developed and optimized, ranging from conventional screening techniques to advanced instrumental approaches. In many cases, a multi-technique strategy is employed to enhance reliability, sensitivity, and structural specificity in glucosinolate analysis [6,55].

The extraction of glucosinolates from plant tissues requires techniques that maintain their stability and prevent their enzymatic degradation. The most employed method is hot hydromethanolic extraction (70–80%), as the immediate application of heat rapidly and effectively inactivates the myrosinase enzyme, whose thermal denaturation typically occurs between 60 °C and 70 °C. Myrosinase is responsible for hydrolyzing glucosinolates into compounds such as isothiocyanates. This approach preserves the original glucosinolate profile and allows efficient recovery of these compounds from the vacuoles of plant cells. Furthermore, the heat facilitates the diffusion of glucosinolates from the plant matrix, ensuring high extraction efficiency [55,56,57]. Extraction methods using cold methanol or ethanol can also inactivate myrosinase; however, the use of solvent mixtures with low alcohol concentration may reduce extraction efficiency and preserve some residual myrosinase activity, causing variability in results. For this reason, hot extraction is generally preferred to ensure consistent inactivation and reliable recovery of glucosinolates. Some protocols employ boiling water to avoid the use of organic solvents; however, this approach may not be suitable for all glucosinolate types, particularly heat-sensitive ones. The thermal stability of glucosinolates is largely determined by their chemical structure. While all GSLs share a common core, the nature of the side chain (R group) influences how rapidly they degrade when exposed to heat. Indolic glucosinolates are the most heat-sensitive, breaking down significantly even at temperatures below 100 °C. Aliphatic glucosinolates are generally more stable under typical cooking conditions (≈100 °C), but at higher temperatures (110–120 °C) differences between classes diminish, and substantial degradation occurs. Aromatic glucosinolates tend to be the most heat-resistant, maintaining greater stability than aliphatic forms during thermal processing.

As an alternative, modern techniques using supercritical fluid extraction (SFE) have been explored. Studies in *Eruca sativa* demonstrated that SFE with co-solvents such as water, methanol, or ethanol allows efficient glucosinolates recovery [58]. The addition of water as a co-solvent proved to be particularly effective, enabling efficient glucosinolate extraction at a lower temperature (45 °C) compared with conventional methods, reducing the need for toxic solvents, and providing a more sustainable approach for food and nutraceutical applications [58]. The choice of extraction method ultimately depends on the type of plant tissue, the class of glucosinolates of interest (aliphatic, aromatic, or indolic), and the balance between yield, compound preservation, operational safety, and environmental impact. However, supercritical fluid extraction of glucosinolates remains poorly studied and requires further investigation [56,57].

For glucosinolate identification, thin-layer chromatography (TLC) has traditionally served as a preliminary tool, especially in exploratory phytochemical studies. Following extraction with hot water or hydroalcoholic solutions, and after inactivation of the myrosinase enzyme by heat treatment to prevent enzymatic degradation, the extracts are applied onto silica gel plates and eluted using polar solvent systems, such as n-butanol–n-propanol–glacial acetic acid–water (30:10:10:10). Visualization is typically achieved using general chromogenic spray reagents, such as anisaldehyde–sulfuric acid or ferric chloride. Anisaldehyde–sulfuric acid reacts primarily with the sulfated oxime group, while ferric chloride targets phenolic groups in aromatic and indolic glucosinolates. These reagents, however, are not specific to glucosinolates, as they are also commonly used to detect other classes of compounds. Consequently, TLC cannot reliably identify novel glucosinolate structures, which is why more advanced analytical techniques such as mass spectrometry and nuclear magnetic resonance (NMR) are essential to complement TLC data, providing detailed structural elucidation. Despite these limitations, TLC remains a valuable tool for rapid screening, monitoring purification steps, and preliminary comparison of glucosinolate profiles [59].

Liquid chromatography coupled to mass spectrometry (LC-MS) represents the most powerful approach for structural identification of glucosinolates in extracts [6,55]. Electrospray ionization in negative mode is most used, producing primarily [M−H]^−^ pseudomolecular ions. LC-MS/MS analysis reveals characteristic fragmentation patterns, including neutral loss of 80 Da corresponding to the sulfate group, formation of the *m*/*z* 97 ion (HSO_4_^−^), and fragments associated with the common thioglucoside core, such as *m*/*z* 259 [3]. Side chain–specific ions enable differentiation among aliphatic, aromatic, and indolic glucosinolates. However, this technique cannot sometimes distinguish isomers that produce similar fragmentation patterns [60]. High-resolution systems, such as QTOF or Orbitrap, provide exact mass measurements with errors of a few ppm, enabling unambiguous molecular formula assignment and detection of minor compounds [6,61]. Metabolomics-based LC-HRMS approaches further expand the understanding of glucosinolate structural diversity, enabling the detection of new compounds and degradation products. Thus, the combination of classical and advanced instrumental techniques represents the current gold standard for glucosinolate analysis, ensuring high sensitivity, specificity, and analytical robustness in research, quality control, and agro-food applications [6,53,54,55,62,63].

In addition to the liquid chromatography techniques previously mentioned for the analysis of glucosinolates, their volatile derivatives can be investigated using gas chromatography with mass spectrometry detection (GC-MS) [61,64,65,66,67,68,69,70]. Isothiocyanates are typically generated through the enzymatic hydrolysis of glucosinolates and extracted from plant material using organic solvents, such as dichloromethane. Alternatively, solid-phase microextraction (SPME) combined with GC-MS has been employed, allowing the direct analysis of volatile compounds without the need for large solvent volumes [61,71,72,73,74,75].

Another analytical technique for structural identification is NMR spectroscopy. Through ^1^H and ^13^C NMR, chemical shifts and coupling constants can be assigned, allowing the elucidation of side chains and functional groups of glucosinolates and their desulfated derivatives, using two-dimensional experiments such as COSY, HSQC, and HMBC [76]. This technique complements mass spectrometry techniques, enabling the identification of isomers and providing detailed structural information. However, it is not suitable for highly complex extracts and is typically applied to isolated compounds [77]. Moreover, NMR has been employed to study the biotransformation of sinigrin and other glucosinolates by intestinal microbiota, identifying specific degradation products [78].

Colorimetric methods are commonly used for the overall quantification of glucosinolates, mainly through indirect approaches [61,79]. A widely used method involves enzymatic hydrolysis by myrosinase, producing isothiocyanates that react with 1,2-benzenedithiol to form a compound measured at 365 nm. Another approach uses alkaline hydrolysis to release thioglucose, which reacts with a ferricyanide reagent and is measured at 420 nm. These methods provide only total glucosinolate content, expressed as equivalents of a standard compound, and do not allow identification of individual glucosinolates [80,81].

For quantification, high-performance liquid chromatography coupled with ultraviolet (HPLC-UV) or photodiode array (HPLC-PDA) detectors is the reference method for the separation and individual quantification of glucosinolates. The determination of glucosinolates in plant materials is typically carried out using HPLC following extraction, purification, and enzymatic desulfation, recognized by international standards for robust and comparable analysis [52,53,54]. Historically, the AOAC Official Method 916.06 was one of the first widely adopted procedures for glucosinolate quantification. The sample extracts are often purified using anion-exchange columns (e.g., DEAE-Sephadex). Glucosinolates are eluted with a potassium chloride (KCl) solution and converted into desulfoglucosinolates using arylsulfatase before chromatographic analysis. Separation is performed on C18 reversed-phase columns using aqueous/acetonitrile mobile phases, with detection by UV at 229 nm. Quantification is carried out using external calibration curves or relative response factors. Despite its widespread use, AOAC 916.06 lacked detailed instructions on column verification, system suitability, sulfatase preparation, and interlaboratory reproducibility, which could compromise result comparability [52].

The ISO 9167-1:1992 [53] provided an international reference method for glucosinolate analysis in rapeseed and rapeseed meal. It followed the AOAC procedure, emphasizing HPLC with desulfation, but it was limited to rapeseed matrices and provided minimal guidance on validation, reagent quality, and performance checks, limiting reproducibility across different laboratories. The current standard, ISO 9167:2019 [54], addresses these gaps and is now the official method. Following purification, the eluates are treated with arylsulfatase to produce desulfoglucosinolates, with detailed guidance provided on sulfatase preparation, reaction time, and verification of complete desulfation. The desulfoglucosinolates are then separated by HPLC on C18 reversed-phase columns using aqueous/acetonitrile gradients, with detection at 229 nm. Each desulfoglucosinolate is identified by comparison of retention times with known standards and quantified using external calibration curves or relative response factors, ensuring high reproducibility and interlaboratory comparability. It also provides guidance for applying the method to other plant matrices, provided that the glucosinolates have been previously identified, and allows the use of isocratic gradients as an alternative under controlled conditions.

Liquid chromatography coupled with mass spectrometry (LC-MS) is also widely applied for quantifying glucosinolates because of its high sensitivity and selectivity. UHPLC-Q-TOF-MS has been used to identify and quantify 13 intact glucosinolates in Chinese cabbage at different growth stages, demonstrating its utility for profiling glucosinolate variation during development [82]. LC-MS/MS methods have been established for the simultaneous analysis of multiple glucosinolates in vegetable extracts and blood plasma, enabling accurate quantification of 7 compounds such as sinigrin, glucoraphanin, and gluconasturtiin [83]. Likewise, UHPLC-MS/MS has been successfully applied to quantify 14 glucosinolates in rapeseed seeds, with high sensitivity and minimal enzymatic degradation [84].

More recently, NMR has been increasingly used for the quantification of glucosinolates in seeds. Solid-state NMR (^13^C CP-MAS) enables direct measurement of glucosinolate content while preserving sample integrity and producing results comparable to traditional liquid chromatography. This non-destructive approach is particularly advantageous for analyzing limited or sensitive plant material [85]. In addition, quantitative ^1^H NMR (qHNMR) methods have been applied to determine the purity of isolated glucosinolates, providing precise and reproducible results through the optimization of key parameters such as relaxation times and the use of suitable internal standards [86]. Together, these NMR-based techniques offer complementary and reliable strategies for both structural characterization and accurate quantification of glucosinolates.

## 5. Anti-Inflammatory Activity of Glucosinolate Derivatives

Medicinal plants have been extensively investigated as sources of bioactive compounds with the ability to modulate inflammatory responses, representing a promising complementary strategy for the prevention and management of chronic inflammatory diseases [87,88,89]. Among these compounds, glucosinolate-derived metabolites, including isothiocyanates, thiocyanates, nitriles, epithionitriles, oxazolidine-thiones, I3C and DIM have attracted particular attention due to their broad biological activities, including anti-inflammatory, antioxidant, and immunomodulatory effects in both cellular and animal models [87,88,89]. In this context, a systematic survey was conducted of plant species reported in the scientific literature as sources of glucosinolates, with particular emphasis on studies evaluating their extracts and fractions for anti-inflammatory potential in vitro and in vivo experimental models (Table 1).

The species included in this survey belong predominantly to the order *Brassicales*, encompassing the families *Brassicaceae*, *Capparaceae*, *Caricaceae*, *Cleomaceae*, *Moringaceae*, and *Tropaeolaceae*. Among these, *Brassicaceae* is clearly the most represented family, accounting for the majority of investigated taxa. Frequently studied species include *Brassica oleracea*, *B. rapa*, *B. juncea*, *Raphanus sativus*, *Eruca sativa*, *Isatis tinctoria*, *Lepidium sativum*, *Nasturtium officinale*, *Armoracia rusticana*, and *Eutrema japonicum*. In cellular models, particularly LPS-stimulated RAW 264.7 and J774A.1 macrophages, treatment resulted in a significant reduction in nitric oxide (NO) production, accompanied by decreased levels of pro-inflammatory cytokines, including tumor necrosis factor-α (TNF-α), interleukin-1β (IL-1β), and interleukin-6 (IL-6). In parallel, many studies reported inhibition of inducible nitric oxide synthase (iNOS) and cyclooxygenase-2 (COX-2) expression, as well as downregulation of the nuclear factor kappa-light-chain-enhancer of activated B cells (NF-κB) signaling pathway and, in some cases, modulation of mitogen-activated protein kinase (MAPK) and Janus kinase/signal transducer and activator of transcription (JAK/STAT) pathways [90,91,92,93,94,95,96,97,98,99,100,101,102,103,104,105,106,107,108,109,110,111,112,113,114,115,116,117,118,119,120,121,122,123,124,125,126,127,128,129,130,131,132,133,134,135,136,137,138,139,140,141,142,143,144,145,146,147,148,149,150].

**Table 1:** Composition and anti-inflammatory activity of extracts from glucosinolate-containing plants.

Species	Family	Identified Glucosinolates	Extract/Fraction/Part	In Vitro Assay	In Vivo Assay	Reference
*Armoracia* *rusticana*	Brassicaceae	Sinigrin	Methanolic extract from roots	LPS-stimulated J774A.1 macrophages: Decreased NO, TNF-α and IL-6 release; downregulated iNOS and COX-2; inhibited NF-κB p65 activation; reduced ROS release; increased HO-1 expression	—	[90]
*Brassica* *juncea*	Brassicaceae	Sinigrin	—	—	—	[91]
		—	Methanolic extract from leaves; fractionated: n-butanol and ethyl acetate	LPS-stimulated mouse peritoneal macrophages: decrease NO production	—	[92]
		—	Hydroethanolic extract (50%) from seeds	—	TPA-induced ear edema (mice), Arachidonic acid-induced ear edema (mice) and Croton oil-induced chronic ear inflammation: Decreases ear thickness, MPO activity, TNF-α and IL-6 expression	[93]
		—	Methanolic extract from seeds	—	Carrageenan-induced paw edema (rats): Reduces paw swelling.	[94]
*Brassica* *nigra*	Brassicaceae	Sinigrin	Methanolic extract from leaves, seeds and stems	—	—	[95]
		—	Ethanolic extract from leaves	Protease inhibition assay: Shows in vitro anti-inflammatory activity	Carrageenan-induced paw edema (rats): indicates moderate anti-inflammatory activity	[96]
*Brassica oleracea*	Brassicaceae	Sulforaphane	Aqueous extract and hydroethanolic extract (80%) from sprouts/leaves	LPS-induced RAW 264.7: Decreases NO, TNF-α and IL-6 production	Carrageenan-induced paw edema (mice); DSS colitis; LPS-induced liver injury: Decreases edema and inflammatory mediators	[97]
		Sulforaphane	Hydromethanolic extract (80%) from florets; fractionated: n-hexane, ethyl acetate, n-butanol, aqueous fractions	LPS-induced RAW 264.7: Decreases NO production; downregulates iNOS, COX-2, TNF-α and IL-1β expression (ethyl acetate fraction).	—	[98]
		Glucoraphanin	Aqueous extract from sprouts/leaves	—	Carrageenan-induced paw edema (mice): Reduces paw swelling and inflammatory response	[99]
		Neoglucobrassicin				
		Glucoiberin				
		Glucobrasicin				
		Glucoerucin				
		4-Methoxyglucobrassicin				
		4-Hydroxyglucobrassicin				
*Brassica* *rapa*	Brassicaceae	—	Hydroethanolic extract (95%) from roots; fractionated: n-hexane and ethyl acetate, ethyl acetate, n-butanol, aqueous fractions	LPS-induced RAW 264.7: Inhibits NF-κB activation; decreases NO, TNF-α and IL-6 production; downregulates iNOS expression (ethyl acetate fraction).	Carrageenan-induced paw edema (rats): Reduces paw swelling and inflammatory mediators (ethyl acetate fraction).	[100]
		—	Aqueous extracts from leaves	LPS-induced RAW 264.7: Suppresses NO production; decreases TNF-α and IL-6; inhibits iNOS and COX-2 expression.	—	[101]
		—	Hydroethanolic extract (80%) from leaves	—	Carrageenan-induced paw edema (mice): Decreases paw edema and inflammatory mediators; dose-dependent anti-inflammatory activity.	[102]
		Gluconasturtiin	Hydroethanolic extract (80%) from roots fractionated: n-hexane, chloroform, ethyl acetate, n-butanol and water fractions	LPS-induced RAW 264.7: Reduces NO and PGE_2_ production; inhibits TNF-α, IL-1β, IL-6; downregulates iNOS, COX-2 and IL-6 gene expression (ethyl acetate fraction).	—	[103]
		—	Methanolic extract of root peel and pulp	—	Carrageenan-induced paw edema (rats): Reduces paw swelling; peel extracts are most effective; decreases TNF-α, IL-6, CRP, RF.	[104]
*Capparis* *spinosa*	Capparaceae	Glucocapparin	Hydromethanolic extract from leaves, flowers and flower buds (caper)	—	—	[105]
		Glucobrassicin				
		4-hydroxyglucobrassicin				
		4-methoxyglucobrassicin				
		Neoglucobrassicin				
		Sinigrin				
		Glucotropaeolin				
		—	Methanolic extract; fractionated: water fraction	CFSE-based proliferation assay in human PBMCs: Decreased IL-17, increased IL-4 expression (water fraction)	—	[106]
		—	Aqueous extract from fruits; fractionated	—	Carrageenan-induced paw edema (mice): Decreased edema (2 fractions)	[107]
		—	Methanolic extract from flower buds	—	Carrageenan-induced paw edema (rats) and air pouch model (mice): Reduced edema, TNF-α, IL-1β, neutrophil migration	[108]
*Carica* *papaya*	Caricaceae	—	Ethanolic extract from leaves	—	Carrageenan-induced paw edema (rats): Reduced paw edema; cotton pellet granuloma: Decreased granuloma formation; formaldehyde-induced arthritis: Reduced persistent edema	[109]
		—	Hydroethanolic extract (96%) from leaves	—	Carrageenan-induced paw edema (rats): Decreases paw edema; dose-dependent anti-inflammatory activity.	[110]
		Glucotropaeolin	Aqueous extracts from seeds	—	—	[111]
		—	Aqueous extracts from seeds	—	Hepatotoxicity induced by CCl_4_ (rats): Inhibits NF-κB activation; decrease TNF-α, IL-6, TGF-β e p53 production	[112]
*Cleome* *gynandra*	Cleomaceae	Glucocapparin	—	—	—	[113]
		—	Hydromethanolic extract (90%) from leaves	—	Adjuvant-induced arthritic rats: Reduced paw edema and restored hematological/biochemical markers	[114]
*Eruca* *sativa*	Brassicaceae	Glucoerucin	Extract of seeds	NSC-34 motor neurons exposed to medium of LPS-induced RAW 264.7: prevented neuronal apoptosis; suppressed TLR4 and COX-2 expression; inhibited NLRP3 inflammasome activation; restored IL-10 expression	—	[115]
*Eutrema* *japonicum*	Brassicaceae	Sinigrin	Hydroetanolic extract (60%) and methanol-acetone-water (3:1:1) from leaves, stems and roots	—	—	[116]
		Glucohesperin				
		Glucoibarin				
		Glucobrassicanapin 4-methoxyglucobrassicin				
		5-hexenyl glucosinolate				
		Glucochlearin				
		Gluconapin				
		Glucoalyssin				
		Glucoputranjivin				
		—	Hydroethanolic extracts (50%) from roots	—	DSS-induced colitis model (mice)	[117]
*Isatis* *tinctoria*	Brassicaceae	Glucobrassicin	Methanolic extract from leaves	—	—	[118]
		—	Hydroethanolic extract (70%) from leaves	LPS-stimulated RAW 264.7: inhibits NF-κB activation; decrease NO, TNF-α, IL-6 production (anti-inflammatory effects)	DSS-induced colitis (mice): reduced colonic inflammation	[119]
		—	Ethanolic extract from seeds	—	Carrageenan-induced paw edema (rats): Reduced paw swelling; normalized inflammatory biomarkers (CRP, fibrinogenic)	[120,121]
		—	Supercritical fluid extraction from root	—	LPS-induced periodontitis (rats): decreased TNF-α, IL-6, IL-1β in the gingival tissue; reduced inflammatory cell infiltration	[122]
*Lepidium* *sativum*	Brassicaceae	Glucotropaeolin	Ethanolic extract from in seed and whole plant	—	—	[123]
		Gluconasturin				
		—	Aqueous extract from seeds	—	Carrageenan- and formaldehyde-induced paw edema (rats): Reduced edema in both acute and chronic models	[124]
		—	Aqueous extract from seeds; fractionated	—	Carrageenan-induced paw edema (mice): Significant reduction of edema.	[125]
*Moringa* *oleifera*	Moringaceae	Glucomoringin	Hydromethanolic extract from stems, leaves and seeds	—	—	[126]
		—	Ethanolic extract from leaves; fractionated: butanol, ethyl acetate, chloroform, and hexane	LPS-induced RAW 264.7 macrophages: Decreased NO, TNF-α, IL-6, IL-1β; downregulated iNOS and COX-2; inhibited NF-κB and MAPK	—	[127]
		—	Hydroethanolic extract (80%) from flowers	LPS-induced RAW 264.7: Decreased NO, TNF-α, IL-6 and IL-1β; suppressed iNOS and COX-2; inhibited NF-κB signaling; increased IκB-α expression	—	[128]
		—	Hydroethanolic extracts from leaves (50, 70 and 90%)	LPS-induced RAW 264.7: Decreased NO, PGE_2_, TNF-α, IL-6 and IL-1β production; downregulated iNOS and COX-2 expression; inhibited NF-κB activation; increased IL-10 expression	—	[129]
		—	Hydromethanolic extract (80%) from roots, leaves and fruits	LPS-induced RAW 264.7: Decreased NO, TNF-α, IL-6 and IL-1β; inhibited NF-κB; increased IκB-α expression (fruits extract)	—	[130]
		—	Hydroethanolic extract (90%) of whole pods	LPS-induced RAW 264.7: Decreased NO, TNF-α, IL-6 and IL-1β; inhibited NF-κB and MAPK	—	[131]
		—	Aqueous extract from leaves	—	Carrageenan and formaldehyde induced paw edema (rats); Cotton pellet-induced granuloma (rats): Reduced edema and granuloma formation in acute and chronic models	[132]
		—	Aqueous extract from roots	—	Carrageenan-induced paw edema (rats): Reduced paw swelling.	[133]
		—	Ethanolic extract from bark	—	Carrageenan and egg albumin induced paw edema (rats): Dose-dependent reduction of edema	[134]
		—	Hydroethanolic extract from seeds; fractionated: chloroform fraction	—	Acetic acid-induced colitis (rats): Decrease colonic inflammation	[135]
		—	Aqueous extracts from leaves	—	Acetic acid-induced colitis (rats): Decrease inflammatory markers	[136]
		—	Hydroethanolic extract from leaves	—	DSS-induced colitis (mice): decrease colonic inflammation	[137]
		—	Aqueous extract from leaves	—	DSS-induced colitis (mice): decrease colonic inflammation	[138]
*Nasturtium* *officinale*	Brassicaceae	Gluconasturtiin	Hydromethanolic extract (70%) from aerial parts	—	—	[139]
		—	Hydroethanolic extract (70%) from aerial parts	—	Carrageenan-induced paw edema (rats); Formalin-induced paw edema (rats); TPA-induced ear edema (mice): Reduced edema and histological inflammation (systemic and topical)	[140]
		—	Aqueous, ethanolic and hydroethanolic extracts from aerial parts	—	Carrageenan-induced paw edema (rats): inhibition of paw edema.	[141]
		—	Hydroethanolic extract (70%) from roots	LPS-induced RAW 264.7: decrease NO, PGE_2_, TNF-α, IL-1β, IL-6; Downregulated iNOS and COX-2 expression; Inhibited JAK2/STAT3 phosphorylation; Activated the NRF2/HO-1 signaling pathway	—	[142]
*Raphanus* *sativus*	Brassicaceae	Glucoraphasatin	Aqueous extract from roots	—	—	[143]
		Glucoraphanin				
		Glucoerucin				
		Glucobrassicin				
		—	Methanolic extract of leaves; fractionated: *n*-hexane, chloroform, ethyl acetate, *n*-butanol, and water fractions	LPS-induced RAW 264.7: suppressed NO production; inhibited iNOS and COX-2 expression; inhibited NF-κB activation (chloroform fraction)	—	[144]
		—	Aqueous extract from seeds	—	Ulcerative colitis models (DSS, TNBS)	[145]
		—	Aqueous extract from seeds; fractionated ethyl acetate, n-hexane, n-butanol and water fractions	LPS-induced RAW 264.7: decreasing NO production, TNF-α and IL-6 levels, downregulating iNOS expression, and inhibiting NF-κB and p38 MAPK signaling (Ethyl acetate fraction)	LPS-induced systemic inflammation model (mice): attenuated inflammatory response and improved survival (Aqueous extract)	[146]
		—	Fresh leaf and root juice	—	Carrageenan-induced paw edema and formalin-induced paw edema (rats): reduced paw swelling.	[147]
		Glucotropaeolin	Aqueous and DMSO extract	LPS-stimulated human PBMCs: decreased TNF-α release; inhibited COX-2 expression; suppressed PGE_2_ and LTB_4_ pathways	—	[148]
*Tropaeolum* *majus*	Tropaeolaceae	Glucotropaeolin	Hydromethanolic extract from leaves and flowers	—	—	[149]
		—	Aqueous and ethanolic extracts from leaves	—	Carrageenan induced paw edema (rats); Histamine induced paw edema (rats) and Cotton pellet granuloma (rats): Decrease edema and granuloma formation	[150]

In in vivo models, Brassicaceae species evaluated in classical anti-inflammatory assays, such as carrageenan-induced paw edema in rats or mice, showed significant reductions in paw edema and systemic inflammatory markers. Models of dextran sulfate sodium (DSS)- or acetic acid-induced colitis, as well as LPS-induced liver inflammation, further confirmed the capacity of these extracts to attenuate inflammatory cell infiltration and the production of pro-inflammatory mediators. In some cases, an increase in heme oxygenase-1 (HO-1) expression and activation of the nuclear factor erythroid 2-related factor 2 (NRF2) pathway was also observed, suggesting an additional mechanism related to cellular antioxidant responses. Species such as *Capparis spinosa* (Capparaceae) demonstrated cytokine modulation in human peripheral blood mononuclear cells (PBMCs) and edema reduction in animal models. *Carica papaya* (Caricaceae) exhibited anti-inflammatory activity in models of acute edema and induced arthritis. *Cleome gynandra* (Cleomaceae) showed beneficial effects in adjuvant-induced arthritis, improving haematological and biochemical parameters. *Moringa oleifera* (Moringaceae) has been extensively studied, showing consistent effects both in vitro and in models of colitis, edema, and granuloma, with clear inhibition of NF-κB, MAPK, and pro-inflammatory mediators. Similarly, *Tropaeolum majus* (Tropaeolaceae) demonstrated reductions in TNF-α release and COX-2 expression in PBMCs, as well as efficacy in both acute and chronic inflammation models [90,91,92,93,94,95,96,97,98,99,100,101,102,103,104,105,106,107,108,109,110,111,112,113,114,115,116,117,118,119,120,121,122,123,124,125,126,127,128,129,130,131,132,133,134,135,136,137,138,139,140,141,142,143,144,145,146,147,148,149,150].

**Figure 5:**
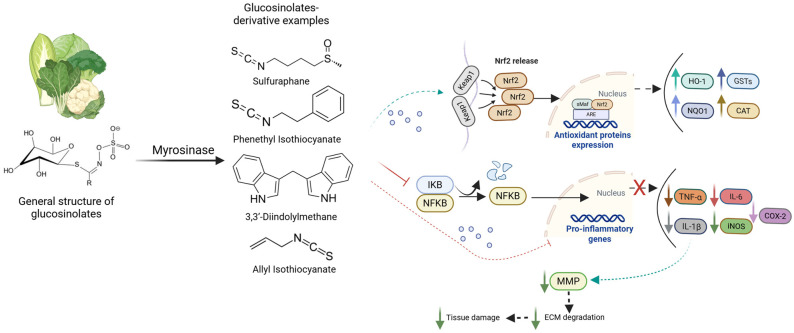
Glucosinolate derivatives and their reported mechanistic effects. Created in BioRender. Ressurreição, S. (2026): https://BioRender.com/65o5m90 (accessed on 10 April 2026).

While the anti-inflammatory effects observed in plant extracts provide important biological evidence, increasing attention has been directed toward identifying the specific bioactive constituents responsible for these activities. In this context, glucosinolate derivatives have been investigated for their anti-inflammatory properties, demonstrating a multifaceted capacity to modulate key molecular pathways implicated in chronic inflammation [151,152]. Table 2 and Figure 5 summarize the main glucosinolates, either isolated from plants or used as commercially available pure compounds, together with their bioactive derivatives. These derivatives are primarily responsible for the anti-inflammatory effects, notably through modulation of the NF-κB and NRF2 pathways, as well as regulation of matrix metalloproteinase (MMP) expression.

A primary mechanism of action of major glucosinolate-derived compounds involves the inhibition of NF-κB nuclear translocation, leading to a marked reduction in the expression of pro-inflammatory cytokines such as TNF-α, IL-6, IL-1β, and IL-8, as well as chemokines and inflammatory enzymes including iNOS and COX-2. This effect has been consistently observed across a variety of in vitro models, including macrophages, epithelial cells, fibroblasts, and cancer cell lines [153,154,155,156,157,158,159,160,161,162,163,164,165,166,167,168,169,170,171,172,173,174,175,176,177,178,179,180,181,182,183,184,185,186,187,188,189,190,191,192,193,194,195,196,197,198,199,200,201,202,203,204,205,206,207,208,209,210,211,212,213,214,215,216,217,218,219,220,221,222,223,224,225,226,227,228,229,230,231,232,233,234,235,236,237,238,239,240,241,242,243,244,245]. It has been corroborated in multiple in vivo models, such as 12-*O*-tetradecanoylphorbol-13-acetate (TPA)-induced edema, lipopolysaccharide (LPS)-induced peritonitis, neuroinflammation, colitis, renal ischemia–reperfusion, and allergic or metabolic inflammatory conditions [207,246,247]. The suppression of NF-κB signaling not only reduces the production of inflammatory mediators but also limits the activation of downstream transcription factors, such as STAT1 and STAT3, attenuating the amplification of inflammatory responses at the transcriptional level [202,248,249].

Concurrently, these derivatives activate the NRF2 signalling pathway, enhancing the expression of cytoprotective and antioxidant enzymes, including HO-1, NAD(P)H:quinone oxidoreductase 1 (NQO1), glutathione S-transferases (GSTs), catalase, superoxide dismutase, and glutamate-cysteine ligase components. This activation contributes to the restoration of cellular redox homeostasis, the replenishment of glutathione levels, the reduction in oxidative stress, and the improvement of mitochondrial function [18,207,249]. The induction of NRF2 target genes has been documented in both in vitro systems and in vivo models, demonstrating consistent enhancement of endogenous defence mechanisms against oxidative damage [207,249]. The dual regulation of NF-κB inhibition and NRF2 activation underscores a coordinated anti-inflammatory and cytoprotective effect, which is crucial for mitigating both acute and chronic inflammatory processes [18,202].

In addition to these canonical pathways, glucosinolate derivatives modulate the activity and expression of MMPs, particularly MMP-2 and MMP-9, contributing to decreased extracellular matrix (ECM) degradation and protection against tissue damage during chronic inflammatory processes. Some derivatives also increase endogenous inhibitors such as Tissue Inhibitor of Metalloproteinases-2 (TIMP-2), suggesting a potential to limit tissue damage while suppressing inflammation [246,249].

Together, these mechanistic effects highlight the therapeutic potential of glucosinolate derivatives while underscoring the need for further exploration of the many glucosinolates that remain insufficiently characterized. In the context of rheumatoid arthritis, chronic synovial inflammation is driven by sustained activation of NF-κB and excessive production of pro-inflammatory cytokines such as TNF-α, IL-1β, and IL-6, which promote pannus formation and progressive cartilage and bone destruction. Experimental evidence indicates that sulforaphane, the bioactive metabolite of glucoraphanin, suppresses NF-κB nuclear translocation and downregulates cytokine expression in immune and synovial cells. Concurrently, it activates the NRF2 pathway, increasing the expression of antioxidant and cytoprotective enzymes, including HO-1 and NQO1 [207,208,209,210,211,212,213,214,215,216,217,218,219,220]. Other glucosinolate-derived isothiocyanates, such as phenethyl isothiocyanate (PEITC), have been shown to reduce the expression and activity of matrix metalloproteinases (MMP-2 and MMP-9), which are directly involved in cartilage degradation [195,196,197,198,199,200,201,202]. These combined anti-inflammatory, antioxidant, and anti-remodeling effects suggest a potential modulatory role in autoimmune joint inflammation, although most available evidence is preclinical.

Metabolic diseases are disorders that arise when the body’s biochemical processes fail to properly convert food into energy [250]. They result from disturbances in metabolic pathways that regulate nutrient processing, storage, and utilization, ultimately leading to impaired physiological function. Examples of these conditions are obesity, type 2 diabetic (T2D) and cardiovascular diseases [251]. Glucosinolate derivatives modulate PI3K/AKT/mTOR, MAPK/JNK, AMPK, PGC 1α, and mitochondrial function, linking anti-inflammatory signaling to improved insulin sensitivity and lipid metabolism, both in vitro studies and in clinical trials, particularly T2D patients [252,253,254,255,256,257]. Sulforaphane and related isothiocyanates also modulate the gut microbiota, promoting the growth of beneficial bacterial taxa and enhancing the biotransformation of glucosinolates into isothiocyanates, thereby further attenuating inflammation and metabolic dysfunction [258,259,260].

In inflammatory bowel disease, which includes ulcerative colitis and Crohn’s disease, persistent activation of NF-κB and elevated levels of TNF-α and IL-6 are central to mucosal injury and epithelial barrier dysfunction [261]. Preclinical studies demonstrate that sulforaphane, 6-methylsulfinylhexyl isothiocyanate (6-MSITC), and benzyl isothiocyanate inhibit NF-κB signaling, decrease COX-2 and iNOS expression, and attenuate oxidative stress through NRF2 activation. These compounds enhance antioxidant defenses by upregulating HO-1, NQO1, glutathione-related enzymes, and other phase II detoxifying proteins [176,177,178,179,180]. Although experimental data are promising, in vivo studies in relevant models remain scarce.

Regarding nonalcoholic fatty liver disease, low-grade chronic inflammation and oxidative stress are key drivers of progression from simple steatosis to steatohepatitis and fibrosis. NF-κB activation in hepatocytes and Kupffer cells enhances pro-inflammatory cytokine production, while impaired antioxidant capacity exacerbates lipid peroxidation and cellular injury. Experimental evidence suggests that sulforaphane (SFN) and allyl isothiocyanate (AITC) suppress NF-κB activation, reduce hepatic TNF-α and IL-6 levels, and stimulate NRF2-dependent antioxidant pathways, restoring enzymes such as HO-1, NQO1, catalase, and glutathione-related systems [207,208,209,210,211,212,213,214,215,216,217,218,219,220,232,233,234,235,236,237,238,239,240,241,242,243]. Some studies also report downregulation of MMP-2 and MMP-9, implicating a potential role in modulating extracellular matrix remodeling and limiting fibrotic progression [262,263]. However, confirmation through large-scale human studies is still required.

In the context of neuroinflammation and related neurodegenerative diseases, chronic activation of microglia and astrocytes results in sustained production of pro-inflammatory mediators and reactive oxygen species, contributing to neuronal dysfunction and degeneration [264,265,266]. Sulforaphane and sulforaphene have been shown to inhibit NF-κB–dependent cytokine production (TNF-α, IL-1β, IL-6) and reduce iNOS expression in experimental models. Simultaneously, activation of Nrf2 enhances antioxidant enzyme expression, including HO-1, NQO1, superoxide dismutase, and catalase, thereby mitigating oxidative neuronal damage [18,207,208,209,210,211,212,213,214,215,216,217,218,219,220,225,227,228,229,230,266,267]. Certain glucosinolate derivatives also reduce MMP-9 expression, which may contribute to preserving blood–brain barrier integrity [252]. Considering the gut–brain axis, microbial metabolism of glucosinolates may further influence systemic and central immune homeostasis, reinforcing the importance of diet–microbiota interactions in neuroimmune regulation [88,268].

Finally, in cancer associated with chronic inflammation, persistent NF-κB signaling promotes tumor initiation, proliferation, angiogenesis, and metastasis. Sustained inflammatory signaling enhances cytokine production and resistance to apoptosis, while MMP-2 and MMP-9 facilitate extracellular matrix degradation and tumor invasion [269,270]. Glucosinolate metabolites such as I3C and DIM, sulforaphane, and BITC inhibit NF-κB signaling, downregulate COX-2 and pro-inflammatory cytokines, and suppress MMP expression in multiple cancer cell models. Concurrent activation of NRF2 strengthens antioxidant defenses, potentially reducing oxidative DNA damage and carcinogenic progression [154,155,156,157,163,164,165,166,167,207,208,209,210,211,212,213,214,215,216,217,218,219,220]. Although these mechanistic findings support a chemopreventive rationale, further clinical validation is necessary to establish their therapeutic relevance in oncology.

Overall, available evidence indicates that glucosinolate derivatives exert biological effects by modulating key inflammatory pathways. Although more than 137 glucosinolates have been identified, only about twelve have been thoroughly studied for the bioactivity of their derivatives, and studies on their anti-inflammatory derivatives remain relatively limited, underscoring a significant gap in current research [32]. The magnitude of these effects critically depends on intestinal microbial conversion and individual bioavailability [20]. Despite encouraging results in experimental models, important gaps remain regarding effective human dosing, interindividual variability, and long-term clinical applicability. Therefore, the interplay between nutrition, gut microbiota, and inflammation represents a promising area of research that requires rigorous clinical investigation to fully establish the therapeutic potential of glucosinolate derivatives.

## 6. Conclusions

Various glucosinolate-rich plants have been investigated, and in cellular models, particularly the widely used LPS-stimulated RAW 264.7 macrophages, these compounds significantly reduced nitric oxide (NO) production, accompanied by decreased levels of pro-inflammatory cytokines, including tumor necrosis factor-α (TNF-α), interleukin-1β (IL-1β), and interleukin-6 (IL-6). In vivo models, classical anti-inflammatory assays, most notably carrageenan-induced paw edema in rats or mice, demonstrated significant reductions in paw edema and systemic inflammatory markers. Additional models, including dextran sulfate sodium (DSS)- or acetic acid-induced colitis and LPS-induced liver inflammation, further confirmed the ability of these extracts to attenuate inflammatory cell infiltration and reduce the production of pro-inflammatory mediators.

Pure or isolated glucosinolate-derived derivatives exhibit significant anti-inflammatory potential through coordinated inhibition of NF-κB, activation of NRF2-mediated antioxidant defenses, and regulation of MMP activity. Although approximately 137 glucosinolates have been identified, only around twelve have been investigated in detail for the bioactivity of their derivatives, highlighting a substantial gap in current research. Evidence from multiple in vitro and in vivo studies consistently shows reductions in pro-inflammatory cytokines, oxidative stress, and MMP-mediated tissue degradation. SFN, I3C/DIM, and AITC have been the most thoroughly investigated glucosinolate-derived compounds.

These findings underscore the therapeutic promise of glucosinolate derivatives in managing inflammation and related pathological conditions, including metabolic disorders, inflammatory bowel disease, nonalcoholic fatty liver disease, low-grade chronic inflammation and oxidative stress, neuroinflammation and related neurodegenerative diseases, as well as cancers linked to chronic inflammation. However, significant research gaps remain, and further studies, including additional in vitro and in vivo experiments as well as well-designed clinical trials, are essential to fully explore their pharmacological potential.

## Figures and Tables

**Table 2:** Biological effects of glucosinolate derivatives on NF-κB, Nrf2, and MMP regulation.

Glucosinolate	Active Derivative	Effect on NF-κB	Effect on NRF2	Effect on MMPs	References
Glucoalyssin	Alyssin	Inhibits NF-κB nuclear translocation: decreases TNF-α, IL-6, IL-8, CCL2, CXCL10 and COX-2. In vitro assays: HPDLCs.	Activates NRF2: increases HO-1 and NQO-1. In vitro assays: HPDLCs.	—	[153]
Glucobrassicin	Indole-3-carbinol (I3C)/3,3′-Diindolylmethane (DIM)	Inhibits NF-κB nuclear translocation: decreases TNF-α, IL-6, IL-1β, iNOS, VEGF and IL-8. In vitro assay: RAW 264.7.	Activates NRF2: increases HO-1, NQO1, GSTs and GCLC/GCLM. In vitro assay: HepG2-C8.	Decreases MMP-9 and uPA. In vitro assay: Prostate cancer cells (PC-3, LNCaP, and C4-2B), breast cancer cells (MCF-7 and MDA-MB-231), pancreatic cancer cells, and squamous cell carcinoma cells. In vivo assay: Xenograft models in SCID/NOD mice, orthotopic pancreatic cancer models, and specialized prostate cancer bone metastasis models.	[154,155,156,157,158,159,160,161,162]
Glucotropaeolin	Benzyl isothiocyanate (BITC)	Inhibits NF-κB nuclear translocation: decreases TNF-α, IL-1β, IL-6, iNOS, COX-2. In vitro assay: RAW 264.7, BV2. In vivo assay: TPA-induced mouse ear edema model and LPS-induced inflammatory response.	Activates NRF2: increases HO-1, NQO1, GSTs and CAT. In vitro assay: RAW 264.7 and Gastric epithelial cell models In vivo assay: Indomethacin-induced gastric injury model	Decreases MMP-2, MMP-9 and uPA; increase TIMP-2. In vitro assay: HT-29 human colon adenocarcinoma cells and SK-Hep1 human hepatocellular carcinoma cells	[163,164,165,166,167,168]
Glucoerucin	Erucin (ER)	Inhibits NF-κB nuclear translocation: decreases TNF-α, IL-1β, IL-6, iNOS, COX-2. In vitro assay: RAW 264.7, HUVEC. In vivo assay: TPA-induced mouse ear edema model and LPS-induced mouse peritonitis model.	Activates NRF2: increases HO-1, NQO1, GSTs and CAT. In vitro assay: HUVEC, TR146, HT-29. In vivo assay: C57BL/6 mice.	—	[169,170,171,172,173,174,175]
Glucohesperin	6-Methylsulfinylhexyl isothiocyanate (6-MSITC)	Inhibits NF-κB signaling via competitive inhibition of GSK-3β: decreases IL-6, CXCL10, and STAT3 activation, and suppresses COX-2, iNOS, and NO production. In vitro assay: TR146 human oral epithelial cells and J774.1/RAW264 macrophages. In vivo assay: DSS-induced colitis models in BALB/c and C57BL/6 mice	Activates NRF2: increases HO-1, NQO1 and GSTs. In vitro assay: TR146 human oral epithelial cells and HepG2. In vivo assay: DSS-induced colitis model in C57BL/6 mice.	—	[176,177,178,179,180,181,182,183,184]
Glucoiberin	Iberin	Inhibits NF-κB nuclear translocation: decreases TNF-α, IL-6 and COX-2. In vitro assay: TNF-α-stimulated TR146 cells. In vivo assay: rat renal ischemia–reperfusion (IRI).	Activates NRF2: increases HO-1 and NQO1. In vitro assay: TR146 cells, fibroblasts NIH3T3.	—	[185,186,187]
Glucomoringin	Moringin (MIC-1)	Inhibits NF-κB nuclear translocation: decreases TNF-α, IL-1β, IL-6, IFN-α, and iNOS. In vitro assay: RAW 264.7, THP-1 and C2C12. In vivo assay: LPS-induced inflammation in C57BL/6 mice.	Activates NRF2: increases HO-1, NQO1 and GSTs. In vitro assay: RAW 264.7 and Hepa1c1c7. In vivo assay: LPS-induced inflammation in C57BL/6 mice.	Decreases MMP-2 and MMP-9. In vitro assay: 786-O and 769-P renal carcinoma cells	[188,189,190,191,192,193,194]
Gluconasturtiin	Phenethyl isothiocyanate (PEITC)	Inhibits NF-κB nuclear translocation: decreases TNF-α, IL-1β, IL-6, IL-8, NO, PGE2, iNOS, COX-2 and CXCL10; reduces STAT1 and STAT3 activation. In vitro assay: RAW 264.7, HMC-1, IPEC-J2, THP-1, HepG2, HT-29, PC-3 and GBM 8401 In vivo assay: C57BL/6J mice, TPA-induced ear edema (ICR mice) and xenograft tumor models	Activates NRF2: increases HO-1, NQO1, SOD, CAT, GSTs, GPx e GR; Restores GSH levels In vitro assay: HepG2, RAW 264.7, primary peritoneal macrophages In vivo assay: diabetic rats (acrylonitrile exposure), C57BL/6 WT vs. Nrf2 knockout	Decreases MMP-2 and MMP-9. In vitro assay: HT-29 colon cancer cells and SAS oral squamous carcinoma cells	[195,196,197,198,199,200,201,202,203,204,205,206]
Glucoraphanin	Sulforaphane (SFN)	Inhibits NF-κB nuclear translocation: decreases TNF-α, IL-1β, IL-6, IL-8, MCP-1, iNOS, COX-2, CXCL10; reduce STAT3 activation; decreases epithelial barrier disruption and inflammation in colitis. In vitro assay: RAW 264.7, HUVEC, TR146 and MCF-7. In vivo assay: BALB/c mice and C57BL/6 mice.	Activates NRF2: increases HO-1, NQO1, GSTs, PGC-1α; Restores GSH levels and mitochondrial DNA copy number. In vitro assay: RAW 264.7, HepG2 and HUVEC. In vivo assay: BALB/c mice and C57BL/6 mice.	Decreases MMP-2 and MMP-9. In vitro assay: U251MG glioblastoma cells and HT-29 colon cancer cells (decrease MMP-2 and MMP-9); MCF-7 breast cancer cells and AGS gastric cancer cells (decrease MMP-9) In vivo assay: LPS-induced acute lung injury model in BALB/c mice (decrease MMP-9 in lung tissue)	[207,208,209,210,211,212,213,214,215,216,217,218,219,220,221,222,223,224]
Glucoraphenin	Sulforaphene	Inhibits NF-κB nuclear translocation: decreases TNF-α, IL-1β, IL-6, IL-8, NO, iNOS, COX-2, and MCP-1 In vitro assay: RAW 264.7, BV-2 microglia, HaCaT and HepG2/Hep3B. In vivo assay: STZ-induced cognitive deficits in rats, D-galactose-induced kidney injury (C57BL/6 mice).	Activates NRF2: increases HO-1, NQO1, GCLM, SOD and CAT. In vitro assay: RAW 264.7, BMMs, HaCaT and HFF. In vivo assay: LPS-induced inflammatory bone erosion (C57BL/6 mice) and D-galactose-induced kidney damage	Decreases MMP-9 (osteoclastogenesis-associated). In vitro assay: RAW 264.7 and BMMs stimulated with RANKL for osteoclastogenesis In vivo assay: In vivo assay: LPS-induced bone erosion model (C57BL/6 mice)	[225,226,227,228,229,230]
Progoitrin	Goitrin	Mild NF-κB inhibition: decreases TNF-α, IL-6 and IL-1β. In vitro assay: RAW 264.7. In vivo assay: C57BL/6J mice.	—	—	[231]
Sinigrin	Allyl isothiocyanate (AITC)	Inhibits NF-κB nuclear translocation: decreases TNF-α, IL-1β, IL-6, iNOS, COX-2. In vitro assay: RAW 264.7, BV2, AML-12, HMC-1, BEAS-2B, HT1376 and THP-1-derived macrophages In vivo assay: C57BL/6 mice: NAFLD/hepatic steatosis (high-fat diet), Traumatic Brain Injury (TBI), BALB/c mice: allergic asthma and Periodontitis model.	Activates NRF2: increases HO-1, NQO1, GSTs and GSH levels. In vitro assay: RAW 264.7, primary astrocytes, fibroblasts In vivo assay: C57BL/6 mice: TBI, NAFLD/steatosis models and BALB/c asthma model	Decreases MMP-2 and MMP-9. In vitro assay: SK-Hep1 hepatoma cells	[232,233,234,235,236,237,238,239,240,241,242,243,244,245]

## Data Availability

The original contributions presented in this study are included in the article. Further inquiries can be directed to the corresponding author.

## Abbreviations

The following abbreviations are used in this manuscript:

^13^C CP-MAS	Carbon-13 Cross-Polarization Magic Angle Spinning
6-MSITC	6-Methylsulfinylhexyl isothiocyanate
AITC	Allyl isothiocyanate
AOAC	Association of Official Analytical Chemists
BALB/c	BALB/c mice
BITC	Benzyl isothiocyanate
BMMs	Bone marrow-derived macrophages
C4-2B	C4-2B prostate cancer cells
C57BL/6	C57BL/6 mice
C57BL/6 WT	C57BL/6 wild-type mice
CAT	Catalase
CCL2	C-C motif chemokine ligand 2
COX-2	Cyclooxygenase-2
COSY	Correlation Spectroscopy
CRP	C-reactive protein
CXCL10	C-X-C motif chemokine ligand 10
DEAE	Diethylaminoethyl
DIM	3,3′-Diindolylmethane
DNA	Deoxyribonucleic acid
DSS	Dextran sulfate sodium
ER	Erucin
ESP	Epithiospecifier protein
GBM 8401	Human glioblastoma cells
GCLM	Glutamate–cysteine ligase modifier subunit
GC-FID	Gas chromatography–flame ionization detection
GC-MS	Gas chromatography–mass spectrometry
GPx	Glutathione peroxidase
GR	Glutathione reductase
GSH	Glutathione
GSK-3β	Glycogen synthase kinase-3 beta
GSTs	Glutathione S-transferases
HaCaT	Human keratinocyte cell line
Hep3B	Human hepatocellular carcinoma cells Hep3B
Hepa1c1c7	Murine hepatoma cells Hepa1c1c7
HepG2	Human hepatocellular carcinoma cells HepG2
HFF	Human foreskin fibroblasts
HMBC	Heteronuclear Multiple Bond Correlation
HMC-1	Human mast cell line-1
HO-1	Heme oxygenase-1
HPDLCs	Human periodontal ligament cells
HPLC	High-performance liquid chromatography
HPLC-PDA	High-performance liquid chromatography with photodiode array detection
HPLC-UV	High-performance liquid chromatography with ultraviolet detection
HSQC	Heteronuclear Single Quantum Coherence
HT-29	Human colorectal adenocarcinoma cells HT-29
I3C	Indole-3-carbinol
ICR	Institute of Cancer Research mice
IL-1β	Interleukin-1 beta
IL-4	Interleukin-4
IL-6	Interleukin-6
IL-8	Interleukin-8
IL-10	Interleukin-10
IL-17	Interleukin-17
iNOS	Inducible nitric oxide synthase
IPEC-J2	Intestinal porcine epithelial cell line J2
J774A.1	Murine macrophage cell line J774A.1
JAK/STAT	Janus kinase/signal transducer and activator of transcription
JAK2/STAT3	Janus kinase 2/signal transducer and activator of transcription 3
LC-HRMS	Liquid chromatography–high-resolution mass spectrometry
LC-MS	Liquid chromatography–mass spectrometry
LC-MS/MS	Liquid chromatography–tandem mass spectrometry
LNCaP	Lymph node carcinoma of the prostate cells
LPS	Lipopolysaccharide
LTB_4_	Leukotriene B4
MAPK	Mitogen-activated protein kinase
MCP-1	Monocyte chemoattractant protein-1
MCF-7	Human breast adenocarcinoma cells MCF-7
MDA-MB-231	Human breast adenocarcinoma cells MDA-MB-231
MIC-1	Moringin
MMP-2	Matrix metalloproteinase 2
MMP-9	Matrix metalloproteinase 9
MMPs	Matrix metalloproteinases
MPO	Myeloperoxidase
MRM	Multiple reaction monitoring
NF-κB	Nuclear factor kappa B
NSC-34	Motor neuron-like cells 34
NSP	Nitrile specifier protein
PBMCs	Peripheral blood mononuclear cells
PC-3	Human prostate cancer cells PC-3
PEITC	Phenethyl isothiocyanate
PGE_2_	Prostaglandin E2
PGC-1α	Peroxisome proliferator-activated receptor gamma coactivator 1-alpha
PLA2	Phospholipase A2
qHNMR	Quantitative proton nuclear magnetic resonance
QTOF	Quadrupole time-of-flight
RANKL	Receptor activator of nuclear factor kappa-B ligand
RAW 264.7	Murine macrophage cell line
RF	Rheumatoid factor
ROS	Reactive oxygen species
SCID/NOD	Severe combined immunodeficiency/non-obese diabetic mice
SFE	Supercritical fluid extraction
SFN	Sulforaphane
SK-Hep1	Human hepatoma cells
SOD	Superoxide dismutase
SPME	Solid-phase microextraction
STAT1	Signal transducer and activator of transcription 1
STAT3	Signal transducer and activator of transcription 3
TFP	Thiocyanate-forming protein
THP-1	Human monocytic leukemia cell line
TIMP-2	Tissue inhibitor of metalloproteinases-2
TLC	Thin-layer chromatography
TLR4	Toll-like receptor 4
TNBS	2,4,6-Trinitrobenzenesulfonic acid
TNF-α	Tumor necrosis factor-alpha
TPA	12-*O*-tetradecanoylphorbol-13-acetate
TR146	Human oral squamous carcinoma cell line TR146
UHPLC-MS/MS	Ultra-high-performance liquid chromatography–tandem mass spectrometry
VEGF	Vascular endothelial growth factor

## References

[1] Vierheilig H., Bennett R., Kiddle G., Kaldorf M., Ludwig-Müller J. (2000). Differences in glucosinolate patterns and arbuscular mycorrhizal status of glucosinolate-containing plant species. New Phytol..

[2] Mithen R., Bennett R., Marquez J. (2010). Glucosinolate biochemical diversity and innovation in the Brassicales. Phytochemistry.

[3] Rodman J.E., Soltis P.S., Soltis D.E., Sytsma K.J., Karol K.G. (1998). Parallel evolution of glucosinolate biosynthesis inferred from congruent nuclear and plastid gene phylogenies. Am. J. Bot..

[4] Pormetter L., Pfalz M., Kagho M.D., Klahn P., Vogel H., Kroymann J., Wittstock U. (2025). Glucosinolate diversity in seven field-collected Brassicaceae species. PLoS ONE.

[5] Daxenbichler M.E., Spencer G.F., Carlson D.G., Rose G.B., Brinker A.M., Powell R.G. (1991). Glucosinolate composition of seeds from 297 species of wild plants. Phytochemistry.

[6] Fahey J.W., Zalcmann A.T., Talalay P. (2001). The chemical diversity and distribution of glucosinolates and isothiocyanates among plants. Phytochemistry.

[7] Halkier B.A., Gershenzon J. (2006). Biology and Biochemistry of Glucosinolates. Annu. Rev. Plant Biol..

[8] Leite P.M., Castilho R.O. (2017). Chemosystematics of Brassicales. Biochem. Syst. Ecol..

[9] Kuchernig J.C., Burow M., Wittstock U. (2012). Evolution of specifier proteins in glucosinolate-containing plants. BMC Evol. Biol..

[10] Kissen R., Bones A.M. (2009). Nitrile-specifier Proteins Involved in Glucosinolate Hydrolysis in *Arabidopsis thaliana*. J. Biol. Chem..

[11] Matusheski N.V., Swarup R., Juvik J.A., Mithen R., Bennett M., Jeffery E.H. (2006). Epithiospecifier Protein from Broccoli (*Brassica oleracea* L. ssp. italica) Inhibits Formation of the Anticancer Agent Sulforaphane. J. Agric. Food Chem..

[12] Matusheski N.V., Juvik J.A., Jeffery E.H. (2004). Heating decreases epithiospecifier protein activity and increases sulforaphane formation in broccoli. Phytochemistry.

[13] Lambrix V., Reichelt M., Mitchell-Olds T., Kliebenstein D.J., Gershenzon J. (2001). The Arabidopsis Epithiospecifier Protein Promotes the Hydrolysis of Glucosinolates to Nitriles and Influences *Trichoplusia ni* Herbivory. Plant Cell.

[14] Kattel S., Antonious G.F., Kattel S., Antonious G.F. (2025). Glucosinolates in Cruciferous Vegetables: Genetic and Environmental Regulation, Metabolic Pathways, and Cancer-Preventive Mechanisms. Int. J. Plant Biol..

[15] Abdel-Massih R.M., Debs E., Othman L., Attieh J., Cabrerizo F.M. (2023). Glucosinolates, a natural chemical arsenal: More to tell than the myrosinase story. Front. Microbiol..

[16] Heiss E., Herhaus C., Klimo K., Bartsch H., Gerhäuser C. (2001). Nuclear Factor κB Is a Molecular Target for Sulforaphane-mediated Anti-inflammatory Mechanisms. J. Biol. Chem..

[17] Folkard D.L., Marlow G., Mithen R.F., Ferguson L.R. (2015). Effect of Sulforaphane on NOD2 via NF-κB: Implications for Crohn’s disease. J. Inflamm..

[18] Habtemariam S. (2024). Anti-Inflammatory Therapeutic Mechanisms of Isothiocyanates: Insights from Sulforaphane. Biomedicines.

[19] Nguyen V.P.T., Stewart J., Lopez M., Ioannou I., Allais F., Nguyen V.P.T., Stewart J., Lopez M., Ioannou I., Allais F. (2020). Glucosinolates: Natural Occurrence, Biosynthesis, Accessibility, Isolation, Structures, and Biological Activities. Molecules.

[20] Sikorska-Zimny K., Beneduce L., Sikorska-Zimny K., Beneduce L. (2021). The Metabolism of Glucosinolates by Gut Microbiota. Nutrients.

[21] Holst B., Williamson G. (2004). A critical review of the bioavailability of glucosinolates and related compounds. Nat. Prod. Rep..

[22] Ressurreição S., Salgueiro L., Figueirinha A. (2025). Diplotaxis muralis as an Emerging Food Crop: Chemical Composition, Nutritional Profile and Antioxidant Activities. Plants.

[23] Ressurreição S., Salgueiro L., Figueirinha A. (2026). Chemical Composition, Nutritional Profile, and Bioactive Properties of Diplotaxis tenuifolia, a Health-Promoting Food. Molecules.

[24] Sikorska-Zimny K., Beneduce L. (2021). The glucosinolates and their bioactive derivatives in Brassica: A review on classification, biosynthesis and content in plant tissues, fate during and after processing, effect on the human organism and interaction with the gut microbiota. Crit. Rev. Food Sci. Nutr..

[25] Radojčić Redovniković I., Glivetić T., Delonga K., Vorkapić-Furač J. (2008). Glucosinolates and their potential role in plant. Period. Biol..

[26] Chhajed S., Mostafa I., He Y., Abou-Hashem M., El-Domiaty M., Chen S. (2020). Glucosinolate Biosynthesis and the Glucosinolate–Myrosinase System in Plant Defense. Agronomy.

[27] Wittstock U., Burow M. (2010). Glucosinolate Breakdown in Arabidopsis: Mechanism, Regulation and Biological Significance. Am. Soc. Plant Biol..

[28] Textor S., Gershenzon J. (2009). Herbivore induction of the glucosinolate–myrosinase defense system: Major trends, biochemical bases and ecological significance. Phytochem. Rev..

[29] Del Carmen Martínez-Ballesta M., Moreno D.A., Carvajal M. (2013). The Physiological Importance of Glucosinolates on Plant Response to Abiotic Stress in Brassica. Int. J. Mol. Sci..

[30] Bones A.M., Rossiter J.T. (1996). The myrosinase-glucosinolate system, its organisation and biochemistry. Physiol. Plant..

[31] Lv Q., Li X., Fan B., Zhu C., Chen Z. (2022). The Cellular and Subcellular Organization of the Glucosinolate–Myrosinase System against Herbivores and Pathogens. Int. J. Mol. Sci..

[32] Ressurreição S., Salgueiro L., Figueirinha A. (2024). Diplotaxis Genus: A Promising Source of Compounds with Nutritional and Biological Properties. Molecules.

[33] Mumm R., Burow M., Bukovinszkine’Kiss G., Kazantzidou E., Wittstock U., Dicke M., Gershenzon J. (2008). Formation of Simple Nitriles upon Glucosinolate Hydrolysis Affects Direct and Indirect Defense Against the Specialist Herbivore, *Pieris rapae*. J. Chem. Ecol..

[34] Ahuja I., de Vos R.C.H., Rohloff J., Stoopen G.M., Halle K.K., Ahmad S.J.N., Hoang L., Hall R.D., Bones A.M. (2016). Arabidopsis myrosinases link the glucosinolate-myrosinase system and the cuticle. Sci. Rep..

[35] Mbudu K.G., Witzel K., Börnke F., Hanschen F.S. (2025). Glucosinolate profile and specifier protein activity determine the glucosinolate hydrolysis product formation in kohlrabi (*Brassica oleracea* var. *gongylodes*) in a tissue-specific way. Food Chem..

[36] Joković N., Pešić S., Vitorović J., Bogdanović A., Sharifi-Rad J., Calina D. (2025). Glucosinolates and Their Hydrolytic Derivatives: Promising Phytochemicals With Anticancer Potential. Phytother. Res..

[37] Aires A., Mota V.R., Saavedra M.J., Rosa E.A.S., Bennett R.N. (2009). The antimicrobial effects of glucosinolates and their respective enzymatic hydrolysis products on bacteria isolated from the human intestinal tract. J. Appl. Microbiol..

[38] Baldelli S., Lombardo M., D’Amato A., Karav S., Tripodi G., Aiello G. (2025). Glucosinolates in Human Health: Metabolic Pathways, Bioavailability, and Potential in Chronic Disease Prevention. Foods.

[39] Fuentes F., Paredes-Gonzalez X., Kong A.-N.T. (2015). Dietary Glucosinolates Sulforaphane, Phenethyl Isothiocyanate, Indole-3-Carbinol/3,3′-Diindolylmethane: Antioxidative Stress/Inflammation, Nrf2, Epigenetics/Epigenomics and In Vivo Cancer Chemopreventive Efficacy. Curr. Pharmacol. Rep..

[40] Kołodziejski D., Koss-Mikołajczyk I., Glatt H., Bartoszek A. (2022). The comparison of cytotoxic and genotoxic activities of glucosinolates, isothiocyanates, and indoles. Sci. Rep..

[41] Srikanth Y., Reddy D.H., Anusha V.L., Dumala N., Viswanadh M.K., Chakravarthi G., Nalluri B.N., Yadagiri G., Ramakrishna K. (2025). Unveiling the Multifaceted Pharmacological Actions of Indole-3-Carbinol and Diindolylmethane: A Comprehensive Review. Plants.

[42] Angelino D., Jeffery E. (2014). Glucosinolate hydrolysis and bioavailability of resulting isothiocyanates: Focus on glucoraphanin. J. Funct. Foods.

[43] Angelino D., Dosz E.B., Sun J., Hoeflinger J.L., Van Tassell M.L., Chen P., Harnly J.M., Miller M.J., Jeffery E.H. (2015). Myrosinase-dependent and–independent formation and control of isothiocyanate products of glucosinolate hydrolysis. Front. Plant Sci..

[44] Fahey J.W., Wehage S.L., Holtzclaw W.D., Kensler T.W., Egner P.A., Shapiro T.A., Talalay P. (2012). Protection of Humans by Plant Glucosinolates: Efficiency of Conversion of Glucosinolates to Isothiocyanates by the Gastrointestinal Microflora. Cancer Prev. Res..

[45] Liou C.S., Sirk S.J., Diaz C.A.C., Klein A.P., Fischer C.R., Higginbottom S.K., Erez A., Donia M.S., Sonnenburg J.L., Sattely E.S. (2020). A Metabolic Pathway for Activation of Dietary Glucosinolates by a Human Gut Symbiont. Cell.

[46] Narra F., Galgani G., Harris C.B., Moreno D.A., Núñez-Gómez V. (2025). Bioavailability, Human Metabolism, and Dietary Interventions of Glucosinolates and Isothiocyanates: Critical Insights and Future Perspectives. Foods.

[47] Dinkova-Kostova A.T., Kostov R.V. (2012). Glucosinolates and isothiocyanates in health and disease. Trends Mol. Med..

[48] Li F., Hullar M.A.J., Beresford S.A.A., Lampe J.W. (2011). Variation of glucoraphanin metabolism in vivo and ex vivo by human gut bacteria. Br. J. Nutr..

[49] Bouranis J.A., Beaver L.M., Ho E. (2021). Metabolic Fate of Dietary Glucosinolates and Their Metabolites: A Role for the Microbiome. Front. Nutr..

[50] Traka M., Mithen R. (2009). Glucosinolates, isothiocyanates and human health. Phytochem. Rev..

[51] Clarke J.D., Dashwood R.H., Ho E. (2008). Multi-targeted prevention of cancer by sulforaphane. Cancer Lett..

[52] Cunniff P. (1995). Official Methods of Analysis of AOAC International.

[53] (1992). Rapeseed—Determination of Glucosinolates Content.

[54] (2019). Rapeseed and Rapeseed Meals–Determination of Glucosinolates Content–Method Using High-Performance Liquid Chromatography.

[55] Clarke D.B. (2010). Glucosinolates, structures and analysis in food. Anal. Methods.

[56] Doheny-Adams T., Redeker K., Kittipol V., Bancroft I., Hartley S.E. (2017). Development of an efficient glucosinolate extraction method. Plant Methods.

[57] Grosser K., Dam N.M. (2017). van A Straightforward Method for Glucosinolate Extraction and Analysis with High-pressure Liquid Chromatography (HPLC). J. Vis. Exp. (JoVE).

[58] Sut S., Boschiero I., Solana M., Malagoli M., Bertucco A., Dall’Acqua S. (2018). Supercritical CO_2_ Extraction of Eruca sativa Using Cosolvents: Phytochemical Composition by LC-MS Analysis. Molecules.

[59] Wagner H., Bladt S. (1996). Plant Drug Analysis: A Thin Layer Chromatography Atlas.

[60] Rochfort S.J., Trenerry V.C., Imsic M., Panozzo J., Jones R. (2008). Class targeted metabolomics: ESI ion trap screening methods for glucosinolates based on MS*^n^* fragmentation. Phytochemistry.

[61] Karanikolopoulou S., Revelou P.-K., Xagoraris M., Kokotou M.G., Constantinou-Kokotou V. (2021). Current Methods for the Extraction and Analysis of Isothiocyanates and Indoles in Cruciferous Vegetables. Analytica.

[62] Cataldi T.R.I., Rubino A., Lelario F., Bufo S.A. (2007). Naturally occurring glucosinolates in plant extracts of rocket salad (*Eruca sativa* L.) identified by liquid chromatography coupled with negative ion electrospray ionization and quadrupole ion-trap mass spectrometry. Rapid Commun. Mass Spectrom..

[63] Palmieri S., Leoni O., Iori R. (1982). A steady-state kineties study of myrosinase with direct ultraviolet spectrophotometric assay. Anal. Biochem..

[64] Sharma A., Rai P.K., Prasad S. (2018). GC–MS detection and determination of major volatile compounds in *Brassica juncea* L. leaves and seeds. Microchem. J..

[65] Klopsch R., Witzel K., Artemyeva A., Ruppel S., Hanschen F.S. (2018). Genotypic Variation of Glucosinolates and Their Breakdown Products in Leaves of *Brassica rapa*. J. Agric. Food Chem..

[66] Zeng W., Tao H., Li Y., Wang J., Xia C., Li S., Wang M., Wang Q., Miao H. (2021). The flavor of Chinese kale sprouts is affected by genotypic variation of glucosinolates and their breakdown products. Food Chem..

[67] Marchioni I., Martinelli M., Ascrizzi R., Gabbrielli C., Flamini G., Pistelli L., Pistelli L. (2021). Small Functional Foods: Comparative Phytochemical and Nutritional Analyses of Five Microgreens of the Brassicaceae Family. Foods.

[68] Ghawi S.K., Methven L., Niranjan K. (2013). The potential to intensify sulforaphane formation in cooked broccoli (*Brassica oleracea var. italica*) using mustard seeds (*Sinapis alba*). Food Chem..

[69] Azizi Naser S., Amiri-Besheli B., Sharifi-Mehr S. (2011). The Isolation and Determination of Sulforaphane from Broccoli Tissues by Reverse Phase-High Performance Liquid Chromatography. J. Chin. Chem. Soc..

[70] Shen L., Su G., Wang X., Du Q., Wang K. (2010). Endogenous and exogenous enzymolysis of vegetable-sourced glucosinolates and influencing factors. Food Chem..

[71] Raffo A., Masci M., Moneta E., Nicoli S., Sánchez del Pulgar J., Paoletti F. (2018). Characterization of volatiles and identification of odor-active compounds of rocket leaves. Food Chem..

[72] Wieczorek M.N., Jeleń H.H. (2019). Volatile Compounds of Selected Raw and Cooked Brassica Vegetables. Molecules.

[73] Wieczorek M.N., Majcher M., Jeleń H. (2020). Comparison of Three Extraction Techniques for the Determination of Volatile Flavor Components in Broccoli. Foods.

[74] Wieczorek M.N., Majcher M.A., Jeleń H.H. (2021). Identification of aroma compounds in raw and cooked broccoli. Flavour Fragr. J..

[75] Wei S., Xiao X., Wei L., Li L., Li G., Liu F., Xie J., Yu J., Zhong Y. (2021). Development and comprehensive HS-SPME/GC–MS analysis optimization, comparison, and evaluation of different cabbage cultivars (*Brassica oleracea* L. var. *capitata* L.) volatile components. Food Chem..

[76] Ibrahim N., Allart-Simon I., De Nicola G.R., Iori R., Renault J.-H., Rollin P., Nuzillard J.-M. (2018). Advanced NMR-Based Structural Investigation of Glucosinolates and Desulfoglucosinolates. J. Nat. Prod..

[77] Borges R.M., Ferreira G.d.A., Campos M.M., Teixeira A.M., Costa F.d.N., das Chagas F.O., Colonna M. (2023). NMR as a tool for compound identification in mixtures. Phytochem. Anal..

[78] Combourieu B., Elfoul L., Delort A.M., Rabot S. (2001). Identification of new derivatives of sinigrin and glucotropaeolin produced by the human digestive microflora using 1H NMR spectroscopy analysis of in vitro incubations. Drug Metab. Dispos..

[79] Zhang Y. (2012). The 1,2-Benzenedithiole-Based Cyclocondensation Assay: A Valuable Tool for the Measurement of Chemopreventive Isothiocyanates. Crit. Rev. Food Sci. Nutr..

[80] Jezek J., Haggett B.G.D., Atkinson A., Rawson D.M. (1999). Determination of Glucosinolates Using Their Alkaline Degradation and Reaction with Ferricyanide. J. Agric. Food Chem..

[81] Gallaher C.M., Gallaher D.D., Peterson S. (2012). Development and Validation of a Spectrophotometric Method for Quantification of Total Glucosinolates in Cruciferous Vegetables. J. Agric. Food Chem..

[82] Zhou B., Huang W., Feng X., Liu Q., Ibrahim S.A., Liu Y. (2022). Identification and quantification of intact glucosinolates at different vegetative growth periods in Chinese cabbage cultivars by UHPLC-Q-TOF-MS. Food Chem..

[83] Song L., Morrison J.J., Botting N.P., Thornalley P.J. (2005). Analysis of glucosinolates, isothiocyanates, and amine degradation products in vegetable extracts and blood plasma by LC–MS/MS. Anal. Biochem..

[84] Yao M., Xing S., Yao G., Yong W., Ling Y., Chu B. (2025). Simultaneous quantification of 14 glucosinolates in rapeseeds by ultra high performance liquid chromatography–Tandem mass spectrometry. Food Chem..

[85] Mazzini S., Zuccolo M., Bassoli A., Gardana C., Borgonovo G. (2025). Quantification of Glucosinolates in Seeds by Solid-State 13C-Nuclear Magnetic Resonance (NMR). Seeds.

[86] Guo Q., Li Z., Shen L., Xiao Y., Cheng Z. (2021). Quantitative 1H nuclear magnetic resonance (qHNMR) methods for accurate purity determination of glucosinolates isolated from *Isatis indigotica* roots. Phytochem. Anal..

[87] Wu J., Cui S., Liu J., Tang X., Zhao J., Zhang H., Mao B., Chen W. (2023). The recent advances of glucosinolates and their metabolites: Metabolism, physiological functions and potential application strategies. Crit. Rev. Food Sci. Nutr..

[88] Muscarà C., Gugliandolo A., Mazzon E., Calì G. (2025). Glucosinolate Metabolites and Brain Health: An Updated Review on Their Potential Benefits in Neurodegenerative, Neurodevelopmental, and Psychiatric Disorders. Antioxidants.

[89] Miękus N., Marszałek K., Podlacha M., Iqbal A., Puchalski C., Świergiel A.H. (2020). Health Benefits of Plant-Derived Sulfur Compounds, Glucosinolates, and Organosulfur Compounds. Molecules.

[90] Marzocco S., Calabrone L., Adesso S., Larocca M., Franceschelli S., Autore G., Martelli G., Rossano R. (2015). Anti-inflammatory activity of horseradish (*Armoracia rusticana*) root extracts in LPS-stimulated macrophages. Food Funct..

[91] Yadav M., Rana J.S. (2018). Quantitative analysis of Sinigrin in Brassica Juncea. J. Pharmacogn. Phytochem..

[92] Kim H.Y., Yokozawa T., Cho E.J. (2005). Mustard Leaf Suppresses Nitric Oxide Synthesis by Mouse Macrophages. J. Nutr. Sci. Vitaminol..

[93] Xian Y.-F., Hu Z., Ip S.-P., Chen J.-N., Su Z.-R., Lai X.-P., Lin Z.-X. (2018). Comparison of the anti-inflammatory effects of *Sinapis alba* and *Brassica juncea* in mouse models of inflammation. Phytomedicine.

[94] Chouhan Y.S., Kataria H.C., Goswami C.S. (2014). Anti-inflammatory activity of methanolic extract of brassica juncea seed on carrageenan induced paw edema in rats. Int. J. Pharm. Sci. Res..

[95] Shahawany A.W.A., Hattab Z.N.A., Tahhan S.F.A. (2017). Qualitative and Quantitative Analysis of Sinigrin in Different Parts *In Vitro* and *In Vivo* of *Brassica nigra* Plants. Biomed. Biotechnol..

[96] Alam M.B., Hossain M.S., Haque M.E. (2011). Antioxidant and anti-inflammatory activities of the leaf extract of brassica nigra. Int. J. Pharm. Sci. Res..

[97] Sim H.W., Lee W.-Y., Lee R., Yang S.Y., Ham Y.-K., Lim S.D., Park H.-J. (2023). The Anti-Inflammatory Effects of Broccoli (*Brassica oleracea* L. var. italica) Sprout Extract in RAW 264.7 Macrophages and a Lipopolysaccharide-Induced Liver Injury Model. Curr. Issues Mol. Biol..

[98] Hwang J.-H., Lim S.-B. (2014). Antioxidant and Anti-inflammatory Activities of Broccoli Florets in LPS-stimulated RAW 264.7 Cells. Prev. Nutr. Food Sci..

[99] Guadarrama-Enríquez O., Moreno-Pérez G.F., González-Trujano M.E., Ángeles-López G.E., Ventura-Martínez R., Díaz-Reval I., Cano-Martínez A., Pellicer F., Baenas N., Moreno D.A. (2023). Antinociceptive and antiedema effects produced in rats by *Brassica oleracea* var. italica sprouts involving sulforaphane. Inflammopharmacology.

[100] Shin J.-S., Yun C.H., Cho Y.-W., Baek N.-I., Choi M.-S., Jeong T.-S., Chung H.-G., Lee K.-T. (2011). Indole-Containing Fractions of Brassica rapa Inhibit Inducible Nitric Oxide Synthase and Pro-Inflammatory Cytokine Expression by Inactivating Nuclear Factor-κB. J. Med. Food.

[101] Choi H., Kim H., Han S., Park H.W., Ha I.J., Kim J.S., Lee S.-G. (2023). Antioxidant and Anti-Inflammatory Activities of High-Glucosinolate-Synthesis Lines of *Brassica rapa*. Antioxidants.

[102] Amin M.N., Hussain F., Islam M.M., Kabir A.K.L., Islam M.M. (2024). In vivo Anti-Inflammatory and Antinociceptive Activity Evaluation of *Brassica Rapa* Ssp. Chinensis Ethanolic Extract with In Vitro Thrombolytic and Anthelmintic Activity Test. Biomed. Pharmacol. J..

[103] Oh B.-M., Oh H.H., Moon K.E., Jang S.-W., Song G.-S. (2024). Antioxidant activity and anti-inflammatory effect of solvent fractions from Chinese cabbage roots (*Brassica rapa* L. ssp. pekinensis). Food Sci. Biotechnol..

[104] Nazar N., Hussain A.I., Rathore H.A. (2024). Inter-Varietal Variation in Phenolic Profile, Antioxidant, Anti-Inflammatory and Analgesic Activities of Two Brassica rapa Varieties: Influence on Pro-Inflammatory Mediators. Molecules.

[105] Maldini M., Foddai M., Natella F., Addis R., Chessa M., Petretto G.L., Tuberoso C.I.G., Pintore G. (2016). Metabolomic study of wild and cultivated caper (*Capparis spinosa* L.) from different areas of Sardinia and their comparative evaluation. J. Mass Spectrom..

[106] Moutia M., El Azhary K., Elouaddari A., Al Jahid A., Jamal Eddine J., Seghrouchni F., Habti N., Badou A. (2016). *Capparis spinosa* L. promotes anti-inflammatory response in vitro through the control of cytokine gene expression in human peripheral blood mononuclear cells. BMC Immunol..

[107] Zhou H., Jian R., Kang J., Huang X., Li Y., Zhuang C., Yang F., Zhang L., Fan X., Wu T. (2010). Anti-inflammatory Effects of Caper (*Capparis spinosa* L.) Fruit Aqueous Extract and the Isolation of Main Phytochemicals. J. Agric. Food Chem..

[108] Kernouf N., Bouriche H., Kada S., Messaoudi D., Assaf A.M., Senator A. (2018). Anti-inflammatory and Immuno-modulatory Effects of *Capparis spinosa* Flower Bud Extract. Annu. Res. Rev. Biol..

[109] Owoyele B.V., Adebukola O.M., Funmilayo A.A., Soladoye A.O. (2008). Anti-inflammatory activities of ethanolic extract of Carica papaya leaves. Inflammopharmacology.

[110] Aulianshah V., Thaharah Y.R., Zakiah N., Handayani R. (2024). Anti-Inflammatory Effect Of Carica Papaya Leaves Extract In Male Wistar Rats Based On Variation Of Concentration. Int. J. Health Pharm. (IJHP).

[111] Nakamura Y., Yoshimoto M., Murata Y., Shimoishi Y., Asai Y., Park E.Y., Sato K., Nakamura Y. (2007). Papaya Seed Represents a Rich Source of Biologically Active Isothiocyanate. J. Agric. Food Chem..

[112] Shaban N.Z., El-Kot S.M., Awad O.M., Hafez A.M., Fouad G.M. (2021). The antioxidant and anti-inflammatory effects of *Carica Papaya* Linn. seeds extract on CCl4-induced liver injury in male rats. BMC Complement Med. Ther..

[113] Mano J., Dicko C., Ouédraogo J.C.W., Bonzi-Coulibaly Y.L. (2025). Glucosinolate profiling in *Cleome gynandra* L. aerial parts based on two extraction methods. Can. J. Chem..

[114] Narendhirakannan R.T., Kandaswamy M., Subramanian S. (2005). Anti-Inflammatory Activity of *Cleome gynandra* L. on Hematological and Cellular Constituents in Adjuvant-Induced Arthritic Rats. J. Med. Food.

[115] Gugliandolo A., Giacoppo S., Ficicchia M., Aliquò A., Bramanti P., Mazzon E. (2018). Eruca sativa seed extract: A novel natural product able to counteract neuroinflammation. Mol. Med. Rep..

[116] Dos Santos Szewczyk K., Skowrońska W., Kruk A., Makuch-Kocka A., Bogucka-Kocka A., Miazga-Karska M., Grzywa-Celińska A., Granica S. (2023). Chemical composition of extracts from leaves, stems and roots of wasabi (*Eutrema japonicum*) and their anti-cancer, anti-inflammatory and anti-microbial activities. Sci. Rep..

[117] Kang J.-H., Choi S., Jang J.-E., Ramalingam P., Ko Y.T., Kim S.Y., Oh S.H. (2017). Wasabia japonica is a potential functional food to prevent colitis via inhibiting the NF-κB signaling pathway. Food Funct..

[118] Mohn T., Plitzko I., Hamburger M. (2009). A comprehensive metabolite profiling of *Isatis tinctoria* leaf extracts. Phytochemistry.

[119] Chung Y.C., Lee A., Jang C.H., Ryuk J.A., Ha H., Hwang Y.-H. (2024). Isatidis Folium Represses Dextran Sulfate Sodium-Induced Colitis and Suppresses the Inflammatory Response by Inhibiting Inflammasome Activation. Nutrients.

[120] Al-Yahya M.A., Mossa J.S., Ageel A.M., Rafatullah S. (1994). Pharmacological and safety evaluation studies on *Lepidium sativum* L., Seeds. Phytomedicine.

[121] Yahla I., Benguiar R., Riazi A. (2021). In vivo anti-inflammatory activity of *Lepidium sativum* (L.) seeds. S. Asian J. Exp. Biol..

[122] Lee S.W., Cho Y.W. (2019). Anti-Inflammatory Effect of the Isatis tinctoria L. Root Extract on Lipopolysaccharide-Induced Periodontitis in Rats. J. Biosci. Med..

[123] Malar M.S.J., Vanmathi S.J., Chairman K. (2018). Phytochemical Analysis of Lepidium Sativum Using UV-VIS and GC-MS. Int. J. Adv. Res..

[124] Raval N.D., Ravishankar B., Ashok B.K. (2013). Anti-inflammatory effect of Chandrashura (*Lepidium sativum* Linn.) an experimental study. AYU Int. Q. J. Res. Ayurveda.

[125] Khan I.U., Jamil Y., Shams F., Farsi S., Humayun M., Hussain A., Ahmad A., Iqbal A., Alrefaei A.F., Ali S. (2024). Unlocking the in vitro and in vivo antioxidant and anti-inflammatory activities of polysaccharide fractions from *Lepidium sativum* seed-coat mucilage. Heliyon.

[126] Amaglo N.K., Bennett R.N., Lo Curto R.B., Rosa E.A.S., Lo Turco V., Giuffrida A., Curto A.L., Crea F., Timpo G.M. (2010). Profiling selected phytochemicals and nutrients in different tissues of the multipurpose tree *Moringa oleifera* L., grown in Ghana. Food Chem..

[127] Arulselvan P., Tan W.S., Gothai S., Muniandy K., Fakurazi S., Esa N.M., Alarfaj A.A., Kumar S.S. (2016). Anti-Inflammatory Potential of Ethyl Acetate Fraction of Moringa oleifera in Downregulating the NF-κB Signaling Pathway in Lipopolysaccharide-Stimulated Macrophages. Molecules.

[128] Tan W.S., Arulselvan P., Karthivashan G., Fakurazi S. (2015). *Moringa oleifera* Flower Extract Suppresses the Activation of Inflammatory Mediators in Lipopolysaccharide-Stimulated RAW 264.7 Macrophages via NF-κB Pathway. Mediat. Inflamm..

[129] Fard M., Arulselvan P., Karthivashan G., Adam S., Fakurazi S. (2015). Bioactive extract from moringa oleifera inhibits the pro-inflammatory mediators in lipopolysaccharide stimulated macrophages. Pharmacogn. Mag..

[130] Lee H.-J., Jeong Y.-J., Lee T.-S., Park Y.-Y., Chae W.-G., Chung I.-K., Chang H.-W., Kim C.-H., Choi Y.-H., Kim W.-J. (2013). Moringa fruit inhibits LPS-induced NO/iNOS expression through suppressing the NF-κ B activation in RAW264.7 cells. Am. J. Chin. Med..

[131] Muangnoi C., Chingsuwanrote P., Praengamthanachoti P., Svasti S., Tuntipopipat S. (2012). *Moringa oleifera* Pod Inhibits Inflammatory Mediator Production by Lipopolysaccharide-Stimulated RAW 264.7 Murine Macrophage Cell Lines. Inflammation.

[132] Mittal A., Sharma M., David A., Vishwakarma P., Saini M., Goel M., Saxena K.K. (2017). An experimental study to evaluate the anti-inflammatory effect of moringa oleifera leaves in animal models. Int. J. Basic Clin. Pharmacol..

[133] Ndiaye M., Dieye A.M., Mariko F., Tall A., Sall Diallo A., Faye B. (2002). Contribution to the study of the anti-inflammatory activity of *Moringa oleifera* (moringaceae). Dakar Med..

[134] Chauhan N., Sharma V., Pareek R., Kushwaha A. (2021). Anti Inflammatory Potential of *Moringa Oleifera* Lam. Bark In Jaipur Region. Asian J. Pharm. Res. Dev..

[135] Minaiyan M., Asghari G., Taheri D., Saeidi M., Nasr-Esfahani S. (2014). Anti-inflammatory effect of *Moringa oleifera* Lam. seeds on acetic acid-induced acute colitis in rats. Avicenna J. Phytomed..

[136] Noubissi P.A., Njilifac Q., Fokam Tagne M.A., Dongmo Nguepi M.S., Foyet Fondjo A., Kouémou Emégam N., Ngakou Mukam J., Zintchem R., Wambe H., Fankem G.O. (2022). Anxiolytic and anti-colitis effects of *Moringa oleifera* leaf-aqueous extract on acetic acid-induced colon inflammation in rat. Biomed. Pharmacother..

[137] Zhang Y., Peng L., Li W., Dai T., Nie L., Xie J., Ai Y., Li L., Tian Y., Sheng J. (2020). Polyphenol Extract of Moringa Oleifera Leaves Alleviates Colonic Inflammation in Dextran Sulfate Sodium-Treated Mice. Evid.-Based Complement. Altern. Med..

[138] Mohamed Husien H., Peng W., Su H., Zhou R., Tao Y., Huang J., Liu M., Bo R., Li J. (2022). *Moringa oleifera* leaf polysaccharide alleviates experimental colitis by inhibiting inflammation and maintaining intestinal barrier. Front. Nutr..

[139] Jeon J., Bong S.J., Park J.S., Park Y.-K., Arasu M.V., Al-Dhabi N.A., Park S.U. (2017). De novo transcriptome analysis and glucosinolate profiling in watercress (*Nasturtium officinale* R. Br.). BMC Genom..

[140] Mostafazadeh M., Sadeghi H., Sadeghi H., Zarezade V., Hadinia A., Panahi Kokhdan E. (2022). Further evidence to support acute and chronic anti-inflammatory effects of *Nasturtium officinale*. Res. Pharm. Sci..

[141] Negi N., Upadhyay S., Rana M. (2023). Phytochemical Investigation, Antioxidant and Anti-inflammatory activity of *Nasturtium officinale* W.T. Aiton. J. Chem. Health Risks.

[142] Jeon H., Yang D., Lee N.H., Ahn M., Kim G. (2020). Inhibitory Effect of Black Radish (*Raphanus sativus* L. var. niger) Extracts on Lipopolysaccharide-Induced Inflammatory Response in the Mouse Monocyte/Macrophage-Like Cell Line RAW 264.7. Prev. Nutr. Food Sci..

[143] Hanlon P.R., Webber D.M., Barnes D.M. (2007). Aqueous Extract from Spanish Black Radish (*Raphanus sativus* L. Var. niger) Induces Detoxification Enzymes in the HepG2 Human Hepatoma Cell Line. J. Agric. Food Chem..

[144] Park H.-J., Song M. (2017). Leaves of Raphanus sativus L. Shows Anti-Inflammatory Activity in LPS-Stimulated Macrophages via Suppression of COX-2 and iNOS Expression. Prev. Nutr. Food Sci..

[145] Choi K.-C., Cho S.-W., Kook S.-H., Chun S.-R., Bhattarai G., Poudel S.B., Kim M.-K., Lee K.-Y., Lee J.-C. (2016). Intestinal anti-inflammatory activity of the seeds of *Raphanus sativus* L. in experimental ulcerative colitis models. J. Ethnopharmacol..

[146] Kook S.-H., Choi K.-C., Lee Y.-H., Cho H.-K., Lee J.-C. (2014). *Raphanus sativus* L. seeds prevent LPS-stimulated inflammatory response through negative regulation of the p38 MAPK-NF-κB pathway. Int. Immunopharmacol..

[147] Kamble S., Ahmed M.Z., Ramabhimaiaha S., Patil P. (2015). Anti-Inflammatory Activity of Raphanus sativus L in Acute and Chronic Experimental Models in Albino Rats. Biomed. Pharmacol. J..

[148] Tran H.T.T., Márton M.-R., Herz C., Maul R., Baldermann S., Schreiner M., Lamy E. (2016). Nasturtium (Indian cress, *Tropaeolum majus nanum*) dually blocks the COX and LOX pathway in primary human immune cells. Phytomedicine.

[149] Aguilera-Angel E.-Y., Ballesteros-Vivas D., Vera-Bravo R., García N., Robles-Camargo J.-E., Costa G.M., Espinal-Ruiz M., Caicedo-Trejos J.P., Camacho A.K.C., Arroyo-Maya I.-J. (2025). Chemical profiling, antioxidant, and antibacterial properties of *Tropaeolum majus* L. extract as a functional food ingredient. Front. Nutr..

[150] Ahmed S.M., Middha A., Omer M., Ramakrishna D. (2015). Analgesic and anti-inflammatory activity of ethanolic and aqueous leaf extract of *Tropaeolum majus* L. Int. J. Pharm..

[151] Medzhitov R., Horng T. (2009). Transcriptional control of the inflammatory response. Nat. Rev. Immunol..

[152] Olayanju J.B., Bozic D., Naidoo U., Sadik O.A. (2024). A Comparative Review of Key Isothiocyanates and Their Health Benefits. Nutrients.

[153] Hosokawa I., Hosokawa Y., Okamoto R., Ozaki K., Hosaka K. (2025). Alyssin Modulates Inflammatory Mediators Expression in Interleukin-1β-Stimulated Human Periodontal Ligament Cells. J. Biochem. Mol. Toxicol..

[154] Cho H.J., Seon M.R., Lee Y.M., Kim J., Kim J.-K., Kim S.G., Park J.H.Y. (2008). 3,3´-Diindolylmethane Suppresses the Inflammatory Response to Lipopolysaccharide in Murine Macrophages123. J. Nutr..

[155] Williams D.E. (2021). Indoles Derived From Glucobrassicin: Cancer Chemoprevention by Indole-3-Carbinol and 3,3’-Diindolylmethane. Front. Nutr..

[156] Li Y., Kong D., Ahmad A., Bao B., Sarkar F.H. (2013). Antioxidant Function of Isoflavone and 3,3′-Diindolylmethane: Are They Important for Cancer Prevention and Therapy?. Antioxid. Redox Signal..

[157] Krajka-Kuźniak V., Szaefer H., Bartoszek A., Baer-Dubowska W. (2011). Modulation of rat hepatic and kidney phase II enzymes by cabbage juices: Comparison with the effects of indole-3-carbinol and phenethyl isothiocyanate. Br. J. Nutr..

[158] Takada Y., Andreeff M., Aggarwal B.B. (2005). Indole-3-carbinol suppresses NF-κB and IκBα kinase activation, causing inhibition of expression of NF-κB-regulated antiapoptotic and metastatic gene products and enhancement of apoptosis in myeloid and leukemia cells. Blood.

[159] Ernst I.M.A., Schuemann C., Wagner A.E., Rimbach G. (2011). 3,3′-Diindolylmethane but not indole-3-carbinol activates Nrf2 and induces Nrf2 target gene expression in cultured murine fibroblasts. Free. Radic. Res..

[160] Saw C.L.-L., Cintrón M., Wu T.-Y., Guo Y., Huang Y., Jeong W.-S., Kong A.-N.T. (2011). Pharmacodynamics of dietary phytochemical indoles I3C and DIM: Induction of Nrf2-mediated phase II drug metabolizing and antioxidant genes and synergism with isothiocyanates. Biopharm. Drug Dispos..

[161] Reyes-Hernández O.D., Figueroa-González G., Quintas-Granados L.I., Gutiérrez-Ruíz S.C., Hernández-Parra H., Romero-Montero A., Del Prado-Audelo M.L., Bernal-Chavez S.A., Cortés H., Peña-Corona S.I. (2023). 3,3′-Diindolylmethane and indole-3-carbinol: Potential therapeutic molecules for cancer chemoprevention and treatment via regulating cellular signaling pathways. Cancer Cell Int..

[162] Yen G.-C., Tsai C.-M., Lu C.-C., Weng C.-J. (2018). Recent progress in natural dietary non-phenolic bioactives on cancers metastasis. J. Food Drug Anal..

[163] Lee Y.M., Seon M.R., Cho H.J., Kim J.-S., Park J.H.Y. (2009). Benzyl isothiocyanate exhibits anti-inflammatory effects in murine macrophages and in mouse skin. J. Mol. Med..

[164] Lai K.-C., Huang A.-C., Hsu S.-C., Kuo C.-L., Yang J.-S., Wu S.-H., Chung J.-G. (2010). Benzyl Isothiocyanate (BITC) Inhibits Migration and Invasion of Human Colon Cancer HT29 Cells by Inhibiting Matrix Metalloproteinase-2/-9 and Urokinase Plasminogen (uPA) through PKC and MAPK Signaling Pathway. J. Agric. Food Chem..

[165] Badawy S.A.E., Ogaly H.A., Abd-Elsalam R.M., Azouz A.A. (2021). Benzyl isothiocyanates modulate inflammation, oxidative stress, and apoptosis via Nrf2/HO-1 and NF-κB signaling pathways on indomethacin-induced gastric injury in rats. Food Funct..

[166] Lee C.-M., Lee D.-S., Jung W.-K., Yoo J.S., Yim M.-J., Choi Y.H., Park S., Seo S.-K., Choi J.S., Lee Y.-M. (2016). Benzyl isothiocyanate inhibits inflammasome activation in E. coli LPS-stimulated BV2 cells. Int. J. Mol. Med..

[167] Chuang W.-T., Yen C.-C., Huang C.-S., Chen H.-W., Lii C.-K. (2020). Benzyl Isothiocyanate Ameliorates High-Fat Diet-Induced Hyperglycemia by Enhancing Nrf2-Dependent Antioxidant Defense-Mediated IRS-1/AKT/TBC1D1 Signaling and GLUT4 Expression in Skeletal Muscle. J. Agric. Food Chem..

[168] Hwang E.-S., Lee H.J. (2008). Benzyl isothiocyanate inhibits metalloproteinase-2/-9 expression by suppressing the mitogen-activated protein kinase in SK-Hep1 human hepatoma cells. Food Chem. Toxicol..

[169] Cho H.J., Lee K.W., Park J.H.Y. (2013). Erucin Exerts Anti-Inflammatory Properties in Murine Macrophages and Mouse Skin: Possible Mediation through the Inhibition of NFκB Signaling. Int. J. Mol. Sci..

[170] Ciccone V., Piragine E., Gorica E., Citi V., Testai L., Pagnotta E., Matteo R., Pecchioni N., Montanaro R., Di Cesare Mannelli L. (2022). Anti-Inflammatory Effect of the Natural H2S-Donor Erucin in Vascular Endothelium. Int. J. Mol. Sci..

[171] Wagner A.E., Sturm C., Piegholdt S., Wolf I.M.A., Esatbeyoglu T., De Nicola G.R., Iori R., Rimbach G. (2015). Myrosinase-treated glucoerucin is a potent inducer of the Nrf2 target gene heme oxygenase 1—Studies in cultured HT-29 cells and mice. J. Nutr. Biochem..

[172] Martelli A., Piragine E., Gorica E., Citi V., Testai L., Pagnotta E., Lazzeri L., Pecchioni N., Ciccone V., Montanaro R. (2021). The H2S-Donor Erucin Exhibits Protective Effects against Vascular Inflammation in Human Endothelial and Smooth Muscle Cells. Antioxidants.

[173] Shimoyama M., Hosokawa Y., Hosokawa I., Ozaki K., Hosaka K. (2024). Effects of erucin on inflammatory mediators and antioxidant enzymes’ expression in TNF-α-stimulated human oral epithelial cells. Immunopharmacol. Immunotoxicol..

[174] Bello I., Smimmo M., d’Emmanuele di Villa Bianca R., Bucci M., Cirino G., Panza E., Brancaleone V. (2023). Erucin, an H2S-Releasing Isothiocyanate, Exerts Anticancer Effects in Human Triple-Negative Breast Cancer Cells Triggering Autophagy-Dependent Apoptotic Cell Death. Int. J. Mol. Sci..

[175] Tarozzi A., Morroni F., Bolondi C., Sita G., Hrelia P., Djemil A., Cantelli-Forti G. (2012). Neuroprotective Effects of Erucin against 6-Hydroxydopamine-Induced Oxidative Damage in a Dopaminergic-like Neuroblastoma Cell Line. Int. J. Mol. Sci..

[176] Shimoyama M., Hosokawa Y., Hosokawa I., Ozaki K., Hosaka K. (2022). 6-(Methylsulfinyl) Hexyl Isothiocyanate Inhibits IL-6 and CXCL10 Production in TNF-α-Stimulated Human Oral Epithelial Cells. Curr. Issues Mol. Biol..

[177] Uto T., Hou D.-X., Morinaga O., Shoyama Y. (2012). Molecular Mechanisms Underlying Anti-Inflammatory Actions of 6-(Methylsulfinyl)hexyl Isothiocyanate Derived from Wasabi (Wasabia japonica). Adv. Pharmacol. Pharm. Sci..

[178] Lohning A., Kidachi Y., Kamiie K., Sasaki K., Ryoyama K., Yamaguchi H. (2021). 6-(methylsulfinyl)hexyl isothiocyanate (6-MITC) from *Wasabia japonica* alleviates inflammatory bowel disease (IBD) by potential inhibition of glycogen synthase kinase 3 beta (GSK-3β). Eur. J. Med. Chem..

[179] Hou D.-X., Korenori Y., Tanigawa S., Yamada-Kato T., Nagai M., He X., He J. (2011). Dynamics of Nrf2 and Keap1 in ARE-Mediated NQO1 Expression by Wasabi 6-(Methylsulfinyl)hexyl Isothiocyanate. J. Agric. Food Chem..

[180] Morroni F., Sita G., Graziosi A., Turrini E., Fimognari C., Tarozzi A., Hrelia P. (2018). Protective Effects of 6-(Methylsulfinyl)hexyl Isothiocyanate on Aβ1-42-Induced Cognitive Deficit, Oxidative Stress, Inflammation, and Apoptosis in Mice. Int. J. Mol. Sci..

[181] Pan X., Xie K., Chen K., He Z., Sakao K., Hou D.-X. (2022). Involvement of AMP-activated Protein Kinase α/Nuclear Factor (Erythroid-derived 2) Like 2-iniatived Signaling Pathway in Cytoprotective Effects of Wasabi 6-(Methylsulfinyl) Hexyl Isothiocyanate. J. Cancer Prev..

[182] Bartkowiak-Wieczorek J., Malesza M., Malesza I., Hadada T., Winkler-Galicki J., Grzelak T., Mądry E. (2024). Methylsulfinyl Hexyl Isothiocyanate (6-MSITC) from Wasabi Is a Promising Candidate for the Treatment of Cancer, Alzheimer’s Disease, and Obesity. Nutrients.

[183] Korenori Y., Tanigawa S., Kumamoto T., Qin S., Daikoku Y., Miyamori K., Nagai M., Hou D.-X. (2013). Modulation of Nrf2/Keap1 system by Wasabi 6-methylthiohexyl isothiocyanate in ARE-mediated NQO1 expression. Mol. Nutr. Food Res..

[184] Yamaguchi H., Kamiie K., Kidachi Y., Noshita T., Umetsu H., Fuke Y., Ryoyama K. (2014). Intracellular accumulation of structurally varied isothiocyanates correlates with inhibition of nitric oxide production in proinflammatory stimuli-activated tumorigenic macrophage-like cells. Bioorganic Med. Chem..

[185] Hosokawa Y., Hosokawa I., Shimoyama M., Fujii A., Sato J., Kadena K., Ozaki K., Hosaka K. (2022). The Anti-Inflammatory Effects of Iberin on TNF-α-Stimulated Human Oral Epithelial Cells: In Vitro Research. Biomedicines.

[186] Ernst I.M.A., Palani K., Esatbeyoglu T., Schwarz K., Rimbach G. (2013). Synthesis and Nrf2-inducing activity of the isothiocyanates iberverin, iberin and cheirolin. Pharmacol. Res..

[187] Yahiya Y.I., Hadi N.R., Raghif A.A., Qassam H., Habooby N.G.S.A. (2023). Role of Iberin as an anti-apoptotic agent on renal ischemia-reperfusion injury in rats. J. Med. Life.

[188] Sailaja B.S., Aita R., Maledatu S., Ribnicky D., Verzi M.P., Raskin I. (2021). Moringa isothiocyanate-1 regulates Nrf2 and NF-κB pathway in response to LPS-driven sepsis and inflammation. PLoS ONE.

[189] Sailaja B.S., Hassan S., Cohen E., Tmenova I., Farias-Pereira R., Verzi M.P., Raskin I. (2022). Moringa isothiocyanate-1 inhibits LPS-induced inflammation in mouse myoblasts and skeletal muscle. PLoS ONE.

[190] Jaja-Chimedza A., Graf B.L., Simmler C., Kim Y., Kuhn P., Pauli G.F., Raskin I. (2017). Biochemical characterization and anti-inflammatory properties of an isothiocyanate-enriched moringa (*Moringa oleifera*) seed extract. PLoS ONE.

[191] Zhang T., Zhao L., Xu M., Jiang P., Zhang K. (2024). Moringin alleviates DSS-induced ulcerative colitis in mice by regulating Nrf2/NF-κB pathway and PI3K/AKT/mTOR pathway. Int. Immunopharmacol..

[192] Cheng D., Gao L., Su S., Sargsyan D., Wu R., Raskin I., Kong A.-N. (2019). Moringa Isothiocyanate Activates Nrf2: Potential Role in Diabetic Nephropathy. AAPS J..

[193] Farias-Pereira R., Camayoc P., Raskin I. (2024). Isothiocyanate-Rich Moringa Seed Extract Activates SKN-1/Nrf2 Pathway in Caenorhabditis elegans. Int. J. Mol. Sci..

[194] Xie J., Qian Y.-Y., Yang Y., Peng L.-J., Mao J.-Y., Yang M.-R., Tian Y., Sheng J. (2022). Isothiocyanate From Moringa oleifera Seeds Inhibits the Growth and Migration of Renal Cancer Cells by Regulating the PTP1B-dependent Src/Ras/Raf/ERK Signaling Pathway. Front. Cell Dev. Biol..

[195] Moon P.-D., Kim H.-M. (2012). Anti-inflammatory effect of phenethyl isothiocyanate, an active ingredient of *Raphanus sativus* Linne. Food Chem..

[196] Dey M., Kuhn P., Ribnicky D., Premkumar V., Reuhl K., Raskin I. (2010). Dietary phenethylisothiocyanate attenuates bowel inflammation in mice. BMC Chem. Biol..

[197] Ma C., Zhang L., Huang Q., Deng Q., Huang F., Xu J. (2025). Phenethyl isothiocyanate ameliorates liver injuries secondary to inflammatory bowel disease. Food Funct..

[198] Boyanapalli S.S.S., Paredes-Gonzalez X., Fuentes F., Zhang C., Guo Y., Pung D., Saw C.L.L., Kong A.-N.T. (2014). Nrf2 Knockout Attenuates the Anti-Inflammatory Effects of Phenethyl Isothiocyanate and Curcumin. Chem. Res. Toxicol..

[199] Park H.-J., Kim S.-J., Park S.-J., Eom S.-H., Gu G.-J., Kim S.H., Youn H.-S. (2013). Phenethyl isothiocyanate regulates inflammation through suppression of the TRIF-dependent signaling pathway of Toll-like receptors. Life Sci..

[200] Lee Y.M., Cho H.J., Ponnuraj S.P., Kim J., Kim J.-S., Kim S.G., Park J.H.Y. (2011). Phenethyl Isothiocyanate Inhibits 12-O-Tetradecanoylphorbol-13-Acetate-Induced Inflammatory Responses in Mouse Skin. J. Med. Food.

[201] Liu S., Lin Z., Mao X., Ge L., Hou L., Le G., Gan F., Wen L., Huang K. (2021). Nontoxic dose of Phenethyl isothiocyanate ameliorates deoxynivalenol-induced cytotoxicity and inflammation in IPEC-J2 cells. Res. Vet. Sci..

[202] Hoch C.C., Shoykhet M., Weiser T., Griesbaum L., Petry J., Hachani K., Multhoff G., Bashiri Dezfouli A., Wollenberg B. (2024). Isothiocyanates in medicine: A comprehensive review on phenylethyl-, allyl-, and benzyl-isothiocyanates. Pharmacol. Res..

[203] Lai K.-C., Hsu S.-C., Kuo C.-L., Ip S.-W., Yang J.-S., Hsu Y.-M., Huang H.-Y., Wu S.-H., Chung J.-G. (2010). Phenethyl Isothiocyanate Inhibited Tumor Migration and Invasion via Suppressing Multiple Signal Transduction Pathways in Human Colon Cancer HT29 Cells. J. Agric. Food Chem..

[204] Chen H.-J., Lin C.-M., Lee C.-Y., Shih N.-C., Amagaya S., Lin Y.-C., Yang J.-S. (2013). Phenethyl isothiocyanate suppresses EGF-stimulated SAS human oral squamous carcinoma cell invasion by targeting EGF receptor signaling. Int. J. Oncol..

[205] Gong A., He M., Krishna Vanaja D., Yin P., Karnes R.J., Young C.Y.F. (2009). Phenethyl isothiocyanate inhibits STAT3 activation in prostate cancer cells. Mol. Nutr. Food Res..

[206] Hsu S.-Y., Lee S.-C., Liu H.-C., Peng S.-F., Chueh F.-S., Lu T.-J., Lee H.-T., Chou Y.-C. (2022). Phenethyl Isothiocyanate Suppresses the Proinflammatory Cytokines in Human Glioblastoma Cells through the PI3K/Akt/NF-κB Signaling Pathway In Vitro. Oxidative Med. Cell. Longev..

[207] Tian Q., Xu Z., Sun Q., Iniguez A.B., Du M., Zhu M.-J. (2022). Broccoli-Derived Glucoraphanin Activates AMPK/PGC1α/NRF2 Pathway and Ameliorates Dextran-Sulphate-Sodium-Induced Colitis in Mice. Antioxidants.

[208] Zhu W., Cremonini E., Mastaloudis A., Oteiza P.I. (2024). Glucoraphanin and sulforaphane mitigate TNFα-induced Caco-2 monolayers permeabilization and inflammation. Redox Biol..

[209] Moon D.-O., Kim M.-O., Kang S.-H., Choi Y.H., Kim G.-Y. (2009). Sulforaphane suppresses TNF-α-mediated activation of NF-κB and induces apoptosis through activation of reactive oxygen species-dependent caspase-3. Cancer Lett..

[210] Lin W., Wu R.T., Wu T., Khor T.-O., Wang H., Kong A.-N. (2008). Sulforaphane suppressed LPS-induced inflammation in mouse peritoneal macrophages through Nrf2 dependent pathway. Biochem. Pharmacol..

[211] Nallasamy P., Si H., Babu P.V.A., Pan D., Fu Y., Brooke E.A.S., Shah H., Zhen W., Zhu H., Liu D. (2014). Sulforaphane reduces vascular inflammation in mice and prevents TNF-α-induced monocyte adhesion to primary endothelial cells through interfering with the NF-κB pathway. J. Nutr. Biochem..

[212] Lee Y.-R., Noh E.-M., Han J.-H., Kim J.-M., Hwang B.-M., Kim B.-S., Jung S.H., Youn H.J., Chung E.Y., Kim J.-S. (2013). Sulforaphane controls TPA-induced MMP-9 expression through the NF-κB signaling pathway, but not AP-1, in MCF-7 breast cancer cells. BMB Rep..

[213] Qi T., Xu F., Yan X., Li S., Li H. (2016). Sulforaphane exerts anti-inflammatory effects against lipopolysaccharide-induced acute lung injury in mice through the Nrf2/ARE pathway. Int. J. Mol. Med..

[214] Ranaweera S.S., Dissanayake C.Y., Natraj P., Lee Y.J., Han C.-H. (2020). Anti-inflammatory effect of sulforaphane on LPS-stimulated RAW 264.7 cells and ob/ob mice. J. Vet. Sci..

[215] Zhou T., Zhou M., Tong C., Zhuo M. (2022). Cauliflower bioactive compound sulforaphane inhibits breast cancer development by suppressing NF-κB/MMP-9 signaling pathway expression. Cell Mol. Biol..

[216] Chen M., Yu G., Wu W., Lei P., Zhang X., Ren J., Jiang X., Yang M., He C. (2026). Sulforaphane attenuates DSS-induced ulcerative colitis via the Nrf2/STAT3 signaling pathway and gut microbiota modulation. Food Funct..

[217] Bose C., Alves I., Singh P., Palade P.T., Carvalho E., Børsheim E., Jun S.-R., Cheema A., Boerma M., Awasthi S. (2020). Sulforaphane prevents age-associated cardiac and muscular dysfunction through Nrf2 signaling. Aging Cell.

[218] Zhang L., Wang S., Zhang Y., Li F., Yu C. (2023). Sulforaphane alleviates lung ischemia-reperfusion injury through activating Nrf-2/HO-1 signaling. Exp. Ther. Med..

[219] Esfandyari S., Aleyasin A., Noroozi Z., Taheri M., Khodarahmian M., Eslami M., Rashidi Z., Amidi F. (2021). The Protective Effect of Sulforaphane against Oxidative Stress through Activation of NRF2/ARE Pathway in Human Granulosa Cells. Cell J. (Yakhteh).

[220] Li S., Khoi P.N., Yin H., Sah D.K., Kim N.-H., Lian S., Jung Y.-D. (2022). Sulforaphane Suppresses the Nicotine-Induced Expression of the Matrix Metalloproteinase-9 via Inhibiting ROS-Mediated AP-1 and NF-κB Signaling in Human Gastric Cancer Cells. Int. J. Mol. Sci..

[221] Zheng Y., Tao S., Lian F., Chau B.T., Chen J., Sun G., Fang D., Lantz R.C., Zhang D.D. (2012). Sulforaphane prevents pulmonary damage in response to inhaled arsenic by activating the Nrf2-defense response. Toxicol. Appl. Pharmacol..

[222] Tafakh M.S., Saidijam M., Ranjbarnejad T., Malih S., Mirzamohammadi S., Najafi R. (2018). Sulforaphane, a Chemopreventive Compound, Inhibits Cyclooxygenase-2 and Microsomal Prostaglandin E Synthase-1 Expression in Human HT-29 Colon Cancer Cells. Cells Tissues Organs.

[223] Zhang Z., Li C., Shang L., Zhang Y., Zou R., Zhan Y., Bi B. (2016). Sulforaphane induces apoptosis and inhibits invasion in U251MG glioblastoma cells. SpringerPlus.

[224] Pagliarani B., Pruccoli L., Chemello C., Zusso M., Fonseca Moreira J.C., Tarozzi A., Gomes H.M. (2026). Exploring the anti-inflammatory activity of sulforaphane metabolites. Pharmacol. Rep..

[225] Zhang G., Jin C., Zhu Y., Fu F., Wang G., Li S. (2020). Sulforaphene inhibits the progression of osteosarcoma via regulating FSTL1/NF-κB pathway. Life Sci..

[226] Zhang B., Liu P., Sheng H., Guo Y., Han Y., Suo L., Yuan Q. (2023). New Insight into the Potential Protective Function of Sulforaphene against ROS−Mediated Oxidative Stress Damage In Vitro and In Vivo. Int. J. Mol. Sci..

[227] Yao H., Du Y., Jiang B., Liao Y., Zhao Y., Yin M., Li T., Sheng Y., Ji Y., Du M. (2023). Sulforaphene suppresses RANKL-induced osteoclastogenesis and LPS-induced bone erosion by activating Nrf2 signaling pathway. Free. Radic. Biol. Med..

[228] Ren K., Li Z., Li Y., Zhang W., Han X. (2017). Sulforaphene enhances radiosensitivity of hepatocellular carcinoma through suppression of the NF-κB pathway. J. Biochem. Mol. Toxicol..

[229] Yang W., Liu Y., Xu Q.-Q., Xian Y.-F., Lin Z.-X. (2020). Sulforaphene Ameliorates Neuroinflammation and Hyperphosphorylated Tau Protein via Regulating the PI3K/Akt/GSK-3β Pathway in Experimental Models of Alzheimer’s Disease. Oxidative Med. Cell. Longev..

[230] Gao L., Du F., Wang J., Zhao Y., Liu J., Cai D., Zhang X., Wang Y., Zhang S. (2021). Examination of the differences between sulforaphane and sulforaphene in colon cancer: A study based on next-generation sequencing. Oncol. Lett..

[231] Ruan D., Yang J., Luo Q., Shi Y., Ding L., Wang Z., Wang R., Yang L. (2023). The Protective Effects of Goitrin on LPS-Induced Septic Shock in C57BL/6J Mice via Caspase-11 Non-Canonical Inflammasome Inhibition. Molecules.

[232] Latronico T., Larocca M., Milella S., Fasano A., Rossano R., Liuzzi G.M. (2021). Neuroprotective potential of isothiocyanates in an in vitro model of neuroinflammation. Inflammopharmacology.

[233] Caglayan B., Kilic E., Dalay A., Altunay S., Tuzcu M., Erten F., Orhan C., Gunal M.Y., Yulug B., Juturu V. (2019). Allyl isothiocyanate attenuates oxidative stress and inflammation by modulating Nrf2/HO-1 and NF-κB pathways in traumatic brain injury in mice. Mol. Biol. Rep..

[234] Wagner A.E., Boesch-Saadatmandi C., Dose J., Schultheiss G., Rimbach G. (2012). Anti-inflammatory potential of allyl-isothiocyanate–role of Nrf2, NF-κB and microRNA-155. J. Cell. Mol. Med..

[235] Subedi L., Venkatesan R., Kim S.Y. (2017). Neuroprotective and Anti-Inflammatory Activities of Allyl Isothiocyanate through Attenuation of JNK/NF-κB/TNF-α Signaling. Int. J. Mol. Sci..

[236] Minato Y., Aoki-Nonaka Y., Lwin H.Y., Ando D., Warita Y., Matsugishi-Nasu A., Hiyoshi T., Takahashi N., Tabeta K. (2025). Allyl isothiocyanate suppressed periodontal tissue destruction in mice via bacteriostatic and anti-inflammatory activities against *Porphyromonas gingivalis*. Arch. Oral Biol..

[237] Chang W.-J., Chen B.-H., Inbaraj B.S., Chien J.-T. (2019). Preparation of allyl isothiocyanate nanoparticles, their anti-inflammatory activity towards RAW 264.7 macrophage cells and anti-proliferative effect on HT1376 bladder cancer cells. J. Sci. Food Agric..

[238] Lai Y.-H., Chiang Y.-F., Huang K.-C., Chen H.-Y., Ali M., Hsia S.-M. (2023). Allyl isothiocyanate mitigates airway inflammation and constriction in a house dust mite-induced allergic asthma model via upregulation of tight junction proteins and the TRPA1 modulation. Biomed. Pharmacother..

[239] Okulicz M., Hertig I., Król E., Szkudelski T. (2022). Effects of Allyl Isothiocyanate on Oxidative and Inflammatory Stress in Type 2 Diabetic Rats. Molecules.

[240] Lin T.-S., Huang W.-N., Yang J.-L., Peng S.-F., Liu K.-C., Chen J.-C., Hsia T.-C., Huang A.-C. (2023). Allyl isothiocyanate inhibits cell migration and invasion in human gastric cancer AGS cells via affecting PI3K/AKT and MAPK signaling pathway in vitro. Environ. Toxicol..

[241] Li C.-X., Gao J.-G., Wan X.-Y., Chen Y., Xu C.-F., Feng Z.-M., Zeng H., Lin Y.-M., Ma H., Xu P. (2019). Allyl isothiocyanate ameliorates lipid accumulation and inflammation in nonalcoholic fatty liver disease via the Sirt1/AMPK and NF-κB signaling pathways. World J. Gastroenterol..

[242] Zhu W.-T., Li C.-H., Dai T.-T., Song Q., Chen Y., Han Z.-L., Sun N.-X., Wang D.-L. (2023). Effect of allyl isothiocyanate on oxidative stress in COPD via the AhR/CYP1A1 and Nrf2/NQO1 pathways and the underlying mechanism. Phytomedicine.

[243] Han N.-R., Park W., Um J.-Y., Kim H.-M., Jeong H.-J. (2011). Allyl isothiocyanate regulates caspase-1/receptor interacting protein-2 expression. Int. Immunopharmacol..

[244] Hwang E.-S., Lee H.J. (2006). Allyl isothiocyanate and its N-acetylcysteine conjugate suppress metastasis via inhibition of invasion, migration, and matrix metalloproteinase-2/-9 activities in SK-Hep 1 human hepatoma cells. Exp. Biol. Med..

[245] Du D., Lan W., Wang H., Huang Q., Wang F. (2025). Deciphering the therapeutic potential of *Sinigrin*: A promising anti-inflammatory agent for chronic disease management. Phytomedicine.

[246] Laurindo L.F., dos Santos A.R.d.O., de Carvalho A.C.A., Bechara M.D., Guiguer E.L., Goulart R.d.A., Vargas Sinatora R., Araújo A.C., Barbalho S.M. (2023). Phytochemicals and Regulation of NF-kB in Inflammatory Bowel Diseases: An Overview of In Vitro and In Vivo Effects. Metabolites.

[247] Ribeiro M., Cardozo L.F., Paiva B.R., Baptista B.G., Fanton S., Alvarenga L., Lima L.S., Britto I., Nakao L.S., Fouque D. (2024). Sulforaphane Supplementation Did Not Modulate NRF2 and NF-kB mRNA Expressions in Hemodialysis Patients. J. Ren. Nutr..

[248] Bahoosh S.R., Shokoohinia Y., Eftekhari M. (2022). Glucosinolates and their hydrolysis products as potential nutraceuticals to combat cytokine storm in SARS-COV-2. DARU J. Pharm. Sci..

[249] Esteve M. (2020). Mechanisms Underlying Biological Effects of Cruciferous Glucosinolate-Derived Isothiocyanates/Indoles: A Focus on Metabolic Syndrome. Front. Nutr..

[250] Clemente-Suárez V.J., Martín-Rodríguez A., Redondo-Flórez L., López-Mora C., Yáñez-Sepúlveda R., Tornero-Aguilera J.F. (2023). New Insights and Potential Therapeutic Interventions in Metabolic Diseases. Int. J. Mol. Sci..

[251] Heindel J.J., Blumberg B., Cave M., Machtinger R., Mantovani A., Mendez M.A., Nadal A., Palanza P., Panzica G., Sargis R. (2017). Metabolism disrupting chemicals and metabolic disorders. Reprod. Toxicol..

[252] Kamal R.M., Abdull Razis A.F., Mohd Sukri N.S., Perimal E.K., Ahmad H., Patrick R., Djedaini-Pilard F., Mazzon E., Rigaud S. (2022). Beneficial Health Effects of Glucosinolates-Derived Isothiocyanates on Cardiovascular and Neurodegenerative Diseases. Molecules.

[253] Lee H.-W., Lee C.G., Rhee D.-K., Um S.H., Pyo S. (2017). Sinigrin inhibits production of inflammatory mediators by suppressing NF-κB/MAPK pathways or NLRP3 inflammasome activation in macrophages. Int. Immunopharmacol..

[254] Piragine E., Flori L., Di Cesare Mannelli L., Ghelardini C., Pagnotta E., Matteo R., Lazzeri L., Martelli A., Miragliotta V., Pirone A. (2021). Eruca sativa Mill. seed extract promotes anti-obesity and hypoglycemic effects in mice fed with a high-fat diet. Phytother. Res..

[255] Ahn M., Kim J., Yang D., Chun J.-Y., Kim G.O., Shin T. (2021). Indole-3-carbinol alleviates carbon tetrachloride-induced liver injury by inhibiting inflammatory response and regulating lipid metabolism. Adv. Tradit. Med. (ADTM).

[256] Axelsson A.S., Tubbs E., Mecham B., Chacko S., Nenonen H.A., Tang Y., Fahey J.W., Derry J.M., Wollheim C.B., Wierup N. (2017). Sulforaphane reduces hepatic glucose production and improves glucose control in patients with type 2 diabetes. Sci. Transl. Med..

[257] Bahadoran Z., Tohidi M., Nazeri P., Mehran M., Azizi F., Mirmiran P. (2012). Effect of broccoli sprouts on insulin resistance in type 2 diabetic patients: A randomized double-blind clinical trial. Int. J. Food Sci. Nutr..

[258] Bouranis J.A., Beaver L.M., Choi J., Wong C.P., Jiang D., Sharpton T.J., Stevens J.F., Ho E. (2021). Composition of the Gut Microbiome Influences Production of Sulforaphane-Nitrile and Iberin-Nitrile from Glucosinolates in Broccoli Sprouts. Nutrients.

[259] Choi Y., Abdelmegeed M.A., Song B.-J. (2018). Preventive effects of indole-3-carbinol against alcohol-induced liver injury in mice via antioxidant, anti-inflammatory, and anti-apoptotic mechanisms: Role of gut-liver-adipose tissue axis. J. Nutr. Biochem..

[260] Cramer J.M., Teran-Garcia M., Jeffery E.H. (2012). Enhancing sulforaphane absorption and excretion in healthy men through the combined consumption of fresh broccoli sprouts and a glucoraphanin-rich powder. Br. J. Nutr..

[261] Mao H., Zhao X., Sun S. (2025). NF-κB in inflammation and cancer. Cell Mol. Immunol..

[262] Wolosowicz M., Prokopiuk S., Kaminski T.W. (2025). Matrix Metalloproteinase-9 (MMP-9) as a Therapeutic Target: Insights into Molecular Pathways and Clinical Applications. Pharmaceutics.

[263] Mirzaie M., Karimi M., Fallah H., Khaksari M., Nazari-Robati M. (2018). Downregulation of Matrix Metalloproteinases 2 and 9 is Involved in the Protective Effect of Trehalose on Spinal Cord Injury. Int. J. Mol. Cell. Med. IJMCM.

[264] Kwon H.S., Koh S.-H. (2020). Neuroinflammation in neurodegenerative disorders: The roles of microglia and astrocytes. Transl. Neurodegener..

[265] Doke R.R., Lamkhade G.J., Vinchurkar K., Singh S. (2024). Demystifying the Role of Neuroinflammatory Mediators as Biomarkers for Diagnosis, Prognosis, and Treatment of Alzheimer’s Disease: A Review. ACS Pharmacol. Transl. Sci..

[266] Desando G., Gambari L., Amore E., Borciani G., Carpentieri S., Grassi F., Grigolo B., Roseti L. (2025). Role of Dietary Glucosinolates and Isothiocyanate Derivatives in Osteoarthritis: Insights from a Narrative Review. Appl. Sci..

[267] Hwang H.J., Kim J.-E., Lee K.W. (2022). Sulforaphene Attenuates Cutibacterium acnes-Induced Inflammation. J. Microbiol. Biotechnol..

[268] Suganya K., Koo B.-S. (2020). Gut–Brain Axis: Role of Gut Microbiota on Neurological Disorders and How Probiotics/Prebiotics Beneficially Modulate Microbial and Immune Pathways to Improve Brain Functions. Int. J. Mol. Sci..

[269] Karin M., Greten F.R. (2005). NF-κB: Linking inflammation and immunity to cancer development and progression. Nat. Rev. Immunol..

[270] Kessenbrock K., Plaks V., Werb Z. (2010). Matrix Metalloproteinases: Regulators of the Tumor Microenvironment. Cell.

